# Evaluating the Role of Neddylation Modifications in Kidney Renal Clear Cell Carcinoma: An Integrated Approach Using Bioinformatics, MLN4924 Dosing Experiments, and RNA Sequencing

**DOI:** 10.3390/ph17050635

**Published:** 2024-05-15

**Authors:** Dequan Liu, Guangzhen Wu, Shijin Wang, Xu Zheng, Xiangyu Che

**Affiliations:** 1 Department of Urology, The First Affiliated Hospital of Dalian Medical University, Dalian 116011, China; liudq@dmu.edu.cn (D.L.); wuguangzhen@firsthosp-dmu.com (G.W.); wangsj01@dmu.edu.cn (S.W.); 2 Department of Cell Biology, College of Basic Medical Science, Dalian Medical University, Dalian 116011, China

**Keywords:** kidney renal clear cell carcinoma, neddylation, GSVA, RNA sequencing, MLN4924, PSMB10

## Abstract

Background: Neddylation, a post-translational modification process, plays a crucial role in various human neoplasms. However, its connection with kidney renal clear cell carcinoma (KIRC) remains under-researched. Methods: We validated the Gene Set Cancer Analysis Lite (GSCALite) platform against The Cancer Genome Atlas (TCGA) database, analyzing 33 cancer types and their link with 17 neddylation-related genes. This included examining copy number variations (CNVs), single nucleotide variations (SNVs), mRNA expression, cellular pathway involvement, and methylation. Using Gene Set Variation Analysis (GSVA), we categorized these genes into three clusters and examined their impact on KIRC patient prognosis, drug responses, immune infiltration, and oncogenic pathways. Afterward, our objective is to identify genes that exhibit overexpression in KIRC and are associated with an adverse prognosis. After pinpointing the specific target gene, we used the specific inhibitor MLN4924 to inhibit the neddylation pathway to conduct RNA sequencing and related in vitro experiments to verify and study the specificity and potential mechanisms related to the target. This approach is geared towards enhancing our understanding of the prognostic importance of neddylation modification in KIRC. Results: We identified significant CNV, SNV, and methylation events in neddylation-related genes across various cancers, with notably higher expression levels observed in KIRC. Cluster analysis revealed a potential trade-off in the interactions among neddylation-related genes, where both high and low levels of gene expression are linked to adverse prognoses. This association is particularly pronounced concerning lymph node involvement, T stage classification, and Fustat score. Simultaneously, our research discovered that PSMB10 exhibits overexpression in KIRC when compared to normal tissues, negatively impacting patient prognosis. Through RNA sequencing and in vitro assays, we confirmed that the inhibition of neddylation modification could play a role in the regulation of various signaling pathways, thereby influencing the prognosis of KIRC. Moreover, our results underscore PSMB10 as a viable target for therapeutic intervention in KIRC, opening up novel pathways for the development of targeted treatment strategies. Conclusion: This study underscores the regulatory function and potential mechanism of neddylation modification on the phenotype of KIRC, identifying PSMB10 as a key regulatory target with a significant role in influencing the prognosis of KIRC.

## 1. Introduction

Neddylation, a reversible post-translational modification, involves the covalent attachment of NEDD8 (neuronal precursor cell-expressed developmentally down-regulated protein 8), a ubiquitin-like protein, to its substrate proteins [1,2]. Being highly conserved across eukaryotes, NEDD8 is prominently expressed within the nucleus, contrasted by its weaker expression in the cytoplasm [3]. Initially, the NEDD8 gene was cloned from a mouse in 1992 [4], and it demonstrated a 59% similarity to ubiquitin and assumed integral roles in eukaryotic cell metabolism [5]. The initial non-cullin substrate of neddylation identified was breast cancer-associated protein 3 (BCA3), which was isolated from yeast [6]. The cullin-RING ligases (CRLs) have since become the most extensively studied substrates of neddylation [5]. The deregulation of CRLs is implicated in various diseases, including but not limited to cancer, neurodegenerative disorders, and viral infections [7,8,9]. CRLs belong to a diverse family of ubiquitin ligases tasked with the degradation and modification of a plethora of proteins via ubiquitination [7,10]. Given that over 600 human genes have been identified as encoding CRL subunits, this ubiquitin ligase family holds a significant degree of control over the cellular proteome, regulating tens of thousands of proteins via the ubiquitin-proteasome system for degradation or modification [11,12]. The Cullin subunit, an essential component of CRLs, has its activity modulated by neddylation modification [13]. A study conducted in 1998 elucidated that cullin protein and NEDD8 were overexpressed in colon cancer cells and leukemia cells [14,15], reinforcing the correlation between neddylation and cancer progression.

**Figure 1:**
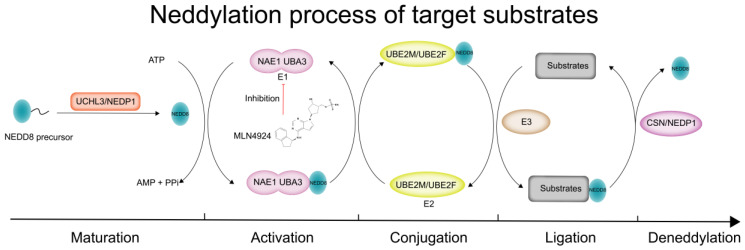
The mechanism diagram illustrates neddylation modification and the targets of MLN4924. NEDD8, neural precursor cell-expressed developmentally down-regulated protein 8; UCH-L3, ubiquitin C-terminal hydrolase-L3; NEDP1, NEDD8 protease 1; NAE1, NEDD8-activating enzyme 1; UBA3, ubiquitin-like modifier-activating enzyme 3; UBE2M/F, ubiquitin-conjugating enzyme E2 M/F; CSN, COP9 signalosome; NEDP1, NEDD8-specific protease 1.

The neddylation process, bearing a resemblance to ubiquitination, encompasses five consecutive enzymatic cascade reactions, detailed as follows (Figure 1):

1. Maturation: The maturation of NEDD8, a crucial process in cellular regulation, begins with the decarboxylation and removal of the C-terminal precursor sequence from NEDD8 precursors. This step is mediated by two key enzymes, NEDD8-specific protease 1 (NEDP1) and ubiquitin C-terminal hydrolase L3 (UCHL3) [2]. NEDP1 plays a specific role in cleaving the C-terminal sequence of NEDD8 precursors, thereby producing active NEDD8 [16]. Concurrently, UCHL3 assists in the elimination of the C-terminal precursor sequence and also contributes to the decarboxylation process during maturation [17]. The activities of these enzymes are vital, as they ensure the proper maturation of NEDD8.
2. Activation: The activation of NEDD8 is carried out by the NEDD8 activating enzyme (NAE), which is composed of two subunits: NAE1 and ubiquitin-like modifier-activating enzyme 3 (UBA3) [18]. In this step, the mature NEDD8 forms a high-energy thioester bond with a cysteine residue within NAE’s active site [19]. This activation of NEDD8 is a critical juncture, as the now active NEDD8 can bind to neddylation substrates [20]. This binding plays a pivotal role in the recognition of CRLs and the ubiquitination of tumor-related proteins [20]. In recent years, MLN4924 has become well-known as a small molecule inhibitor specifically targeting the neddylation pathway [21]. It inhibits the NAE, which is essential for the neddylation process [21]. By inhibiting NAE, MLN4924 effectively blocks the neddylation of cullin proteins, leading to the inactivation of CRLs [22]. This approach highlights the potential of targeting post-translational modification systems in the development of new cancer treatments.
3. Conjugation: Once NEDD8 is activated, it is loaded onto the NAE, setting the stage for its transfer to the neddylation E2-conjugating enzymes, specifically ubiquitin-conjugating enzyme E2 M (UBE2M) and UBE2F [23]. This transfer is facilitated by a trans-thiolation reaction, a critical biochemical mechanism that effectively moves NEDD8 from NAE to the E2 enzyme [24,25]. This step ensures the proper positioning and readiness of NEDD8 for subsequent steps in the protein modification process.
4. Ligation: In the substrate neddylation phase, the final step involves a substrate-specific E3 ligase (either RING-box protein 1/2 (RBX1/2) or DCN1), which plays a crucial role in transferring NEDD8 from the E2 enzyme to the substrate protein [5,26]. This intricate process leads to the formation of a covalent bond between NEDD8 and a lysine residue on the target protein, effectively completing the neddylation process [26]. This step is essential for the regulation of protein function and stability within the cell.
5. Deneddylation: Deneddylation entails the removal of NEDD8 from its substrates [27]. This process is primarily mediated by two key components: the eight-subunit COP9 signalosome (CSN) and NEDD8-specific protease 1 (NEDP1) [28,29,30].

The NEDD8 modification and the subsequent neddylation cascade represent essential intracellular regulatory pathways, which serve crucial roles in a myriad of biological processes within cells, including but not limited to DNA repair, transcriptional regulation, and cell cycle progression [5]. Neddylation, in these contexts, modulates the function of several cellular mechanisms via substrate-specific modification, encompassing cellular proliferation, apoptosis, and signal transduction [5]. Studies have pointed towards a heightened expression of enzymes linked with the neddylation cascade and NEDD8 modification in human cancers [31,32,33,34,35]. Such upregulation is intimately associated with cancer progression and correlated with decreased patient survival rates [31,32,33,34]. Consequently, these enzymes have emerged as significant targets in cancer therapeutics [13]. MLN4924 targets the neddylation process by inhibiting NAE and emerges as a promising contender for cancer therapy, attributed to its extensive anticancer activity and capability to augment the effectiveness of current treatments [36]. Current clinical trials are investigating the efficacy and safety of MLN4924, underlining its therapeutic potential in possibly surmounting drug resistance and selectively targeting cancer cells [37,38,39]. 

Renal cell carcinoma (RCC) is a malignancy originating in the cells lining the kidney’s tubules. Over the years, both its incidence and mortality rate have been on an uptrend, often associated with significant morbidity and mortality [40,41]. RCC is a heterogeneous disease comprising various subtypes, including KIRC, papillary RCC, and chromophobe RCC [42,43]. The most prevalent subtype is KIRC, which accounts for 70–80% of all RCC cases [44,45]. Symptoms of the disease remain largely unnoticeable during the initial stages, but as the condition deteriorates, patients may experience symptoms of varying degrees, such as abdominal pain, hematuria, back pain, and weight loss [46]. The prognosis of KIRC is generally poor, primarily due to its high invasiveness and metastatic capacity [47,48]. Finding new therapeutic targets is the key to current research.

**Figure 2:**
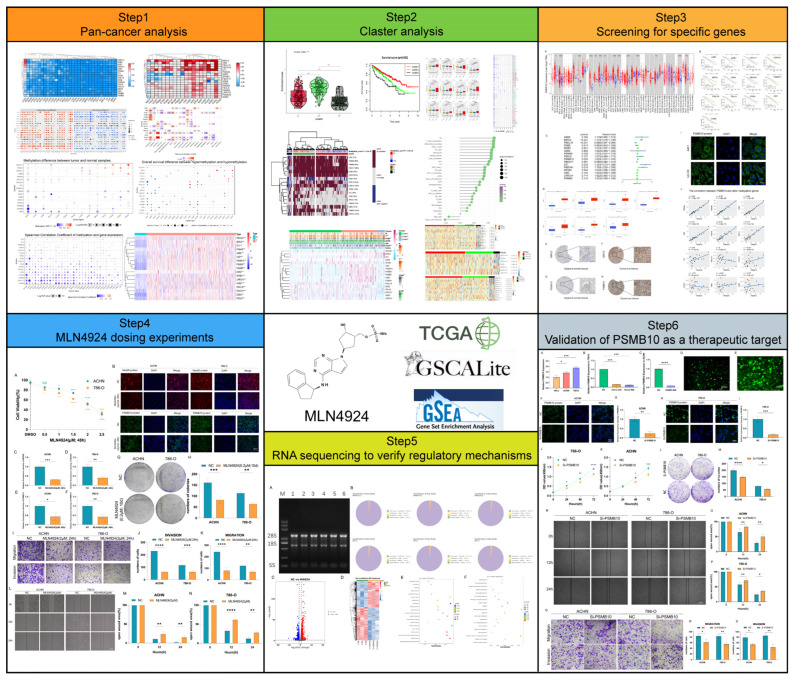
Flowchart of the entire article.

Our study presents an analysis of 17 neddylation-related genes, embodying a wide spectrum of biological functions, notably in protein degradation and ubiquitination exemplified by PSMB8, PSMB9, PSMB10, F-box protein 41 (FBXO41), FBXO17, F-box and leucine-rich repeat protein 16 (FBXL16), FBXL8 [49,50,51,52,53,54,55], cell cycle regulation and apoptosis including baculoviral IAP repeat containing 5 (BIRC5), denticleless E3 ubiquitin protein ligase homolog (DTL) [56,57], immune response and inflammation mediated by PSMB8, PSMB9, PSMB10, and ubiquitin D (UBD) [58,59,60,61], as well as DNA repair and genomic stability with a pivotal contribution from DNA damage-binding protein 2 (DDB2) [62]. Additionally, they are instrumental in signal transduction processes, particularly involving WD repeat and SOCS box containing 1 (WSB1) and the ankyrin repeat and SOCS box protein (ASB) protein family [63,64]. The dysregulation of these critical cellular pathways precipitates a series of events that catalyze tumor initiation and progression in KIRC. This cascade includes uncontrolled cellular proliferation, evasion of programmed cell death, alterations in the tumor microenvironment, and circumvention of immune surveillance. We will conduct bioinformatics analysis and cell experiments on these genes to understand the clinical significance of neddylation-related genes for KIRC and screen out specific genes to provide directions for targeted therapy. A flowchart has been developed to better illustrate our experimental approach (Figure 2).

## 2. Results

### 2.1. Prevalent Mutations in Neddylation-Related Genes

**Figure 3:**
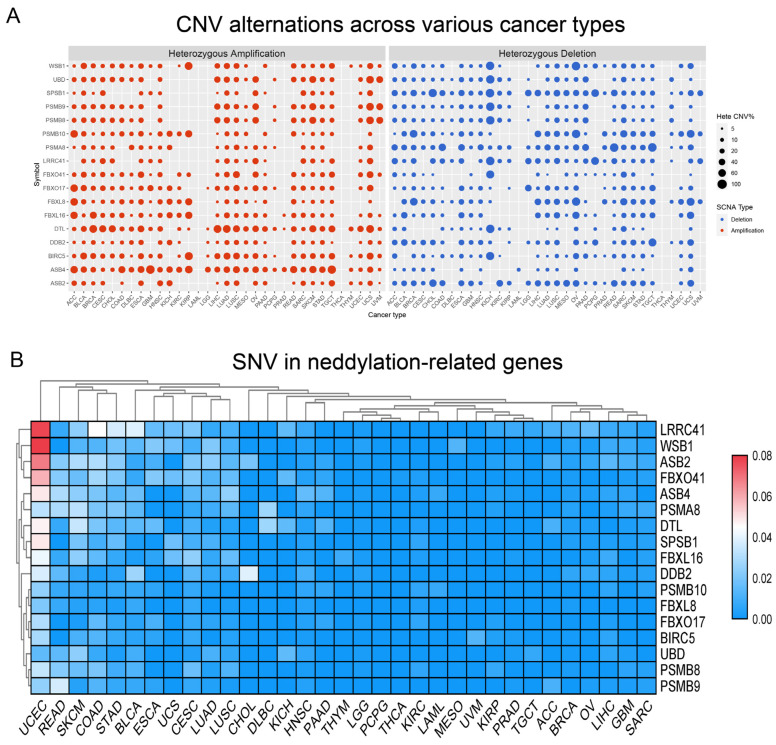
Widespread genetic mutations in neddylation-related genes. (**A**) Solid spheres denote CNV alterations across various cancer types, with the size of the spheres corresponding to the degree of correlation; larger spheres signify higher statistical significance. (**B**) Adjacent color blocks representing SNV within these genes use red to denote high frequency and blue to indicate low frequency.

Our analysis indicated that the majority of cancer types present with CNV and SNV in genes implicated in the neddylation pathway. Interestingly, acute myeloid leukemia (LAML) and thyroid carcinoma (THCA) deviate from other cancer types, displaying minimal CNV in neddylation pathway-related genes. The genes DTL and ASB4 are consistently high in CNV across various cancer types. In contrast, we identified that genes splA/Ryanodine receptor domain and SOCS box containing 1 (SPSB1) and proteasome 20S subunit alpha 8 (PSMA8) are frequently subjected to low CNV in numerous cancer types (Figure 3A). Among the different cancer types, uterine corpus endometrial carcinoma (UCEC) exhibits a heightened frequency of SNVs, whereas lower frequencies are noted in brain lower-grade glioma (LGG), pheochromocytoma and paraganglioma (PCPG), THCA, and sarcoma (SARC) groups (Figure 3B). Our study found that the expression level of neddylation genes in KIRC is higher than that of other cancers (Figure S1A). In addition, our study reveals that neddylation-related genes have a correlation with apoptosis, cell cycle, epithelial-mesenchymal transition (EMT), and hormone ER. These findings are of great significance for the direction of subsequent research (Figure S1B).

### 2.2. Neddylation-Related Genes: The Association and Clinical Significance between Methylation and KIRC

**Figure 4:**
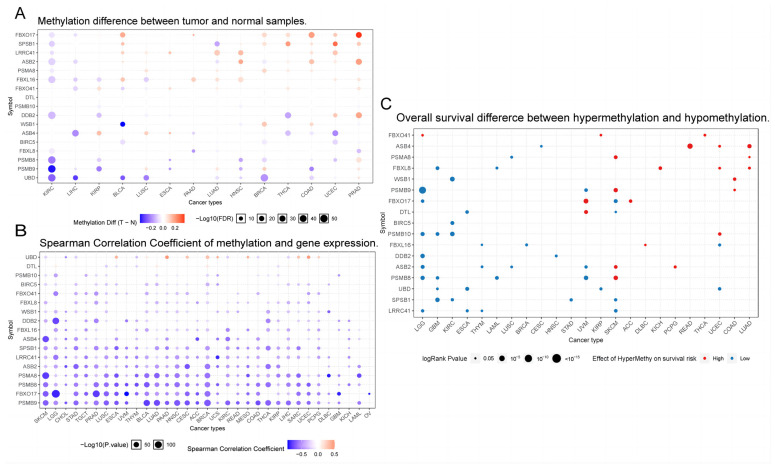
Neddylation-related gene methylation plays a pivotal role in cancer development and progression. In statistical analysis, the correlation between variables measures their mutual relationship. The size of each solid sphere represents the correlation degree between studied variables (A larger sphere signifies a stronger correlation, whereas a smaller sphere indicates a weaker one). (**A**) The figure shows the methylation status of neddylation-related genes across 14 different types of cancers. Higher −log10 (FDR) values indicate greater statistical significance. (**B**) The diagram illustrates the correlation between neddylation-related gene methylation and mRNA expression across 33 types of cancers. The color coding (red for positive correlation and blue for negative) indicates the direction of the correlation. (**C**) This panel displays the association between the methylation status of neddylation-related genes and survival risk across 23 different types of cancers. The survival risk level is represented by a spherical shape, where red indicates a higher risk of mortality, and blue signifies a lower risk.

Our primary focus revolved around the methylation profile of neddylation-related genes and their association with 14 diverse types of cancer (Figure 4A). Our findings illustrate that neddylation-related genes undergo extensive DNA methylation modifications in KIRC. Further, our correlation analysis between methylation of neddylation-related genes and mRNA expression across various cancers unveiled a potent inverse correlation. Predominantly, augmented methylation of neddylation-related genes corresponds to reduced mRNA expression (Figure 4B). However, UBD methylation seems to exhibit a positive influence on mRNA expression. Lastly, we examined the correlation between neddylation-related gene methylation and survival rates across multiple cancer types (Figure 4C). We discovered that methylation of SPSB1, PSMB10, BIRC5, and WSB1 might exert potential protective effects in KIRC. These revelations contribute novel insights towards targeted therapy, and subsequent investigations will aim to ascertain the specificity of these targets.

### 2.3. Impact of Neddylation Pathway Scores on Prognosis in KIRC

**Figure 5:**
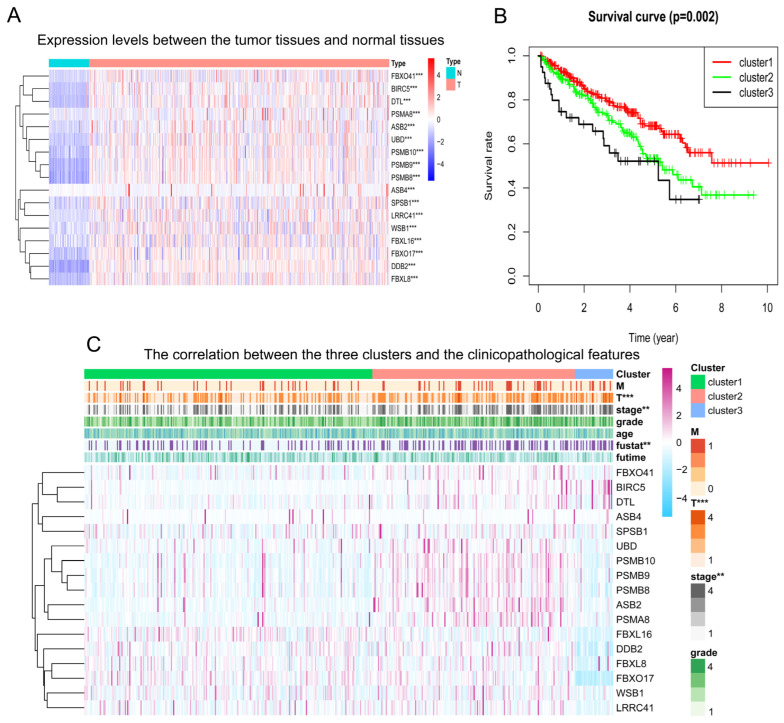
Comprehensive cluster analysis conducted predicated on neddylation scores. (**A**) The illustrated heatmap delineates differential expression of neddylation-related genes between normal and KIRC tissues. Upregulation of gene expression is represented by a red color, while a blue color symbolizes down-regulation. “N” and “T” designate normal and tumor samples, respectively. (**B**) The survival curve delineates disparities in survival probabilities across the three clusters of patients. Red, green, and black lines symbolize Cluster 1, Cluster 2, and Cluster 3, respectively. The horizontal axis signifies years, and the vertical axis stands for survival probability. (**C**) The heatmap delineates the correlation between the three clusters and clinical pathological features, inclusive of M, T, stage, grade, age, Fustat, and futime. Levels of statistical significance are denoted as follows: ** *p* < 0.01, and *** *p* < 0.001.

To explore the role of neddylation in KIRC, we analyzed the gene expression profiles of neddylation-related genes in KIRC tissues compared to normal kidney tissues. Comparative analysis of gene transcription levels between cancerous and normal tissues demonstrated upregulation of these genes in KIRC tissues, underscoring transcriptional disparities, as depicted in the heatmap (Figure 5A). We classified neddylation-related genes into three distinct clusters (C1, C2, and C3) based on the transcriptional differences. Each cluster is defined by unique neddylation pathway scores. The scores aggregate the expression levels of multiple neddylation-related genes within each cluster (Figure S2A). Enrichment analysis further elucidated the distinctions among these clusters, revealing a hierarchy in enrichment scores: C2 > C1 > C3, as shown in the violin plot (Figure S2B).

Survival analysis using Kaplan–Meier curves demonstrated that patients in cluster C1 exhibit a higher overall survival (OS) rate compared with clusters C2 and C3, with C3 showing the lowest OS (Figure 5B). These findings suggest that an imbalance in neddylation-related gene expression, particularly with a predominance of genes with low neddylation scores, is associated with poorer patient prognosis in KIRC. Further analysis revealed a correlation between expression changes of neddylation-related genes and advanced clinical parameters in KIRC, such as increased T stage, overall stage classification, and Fustat score (Figure 5C). This correlation underscores the potential of neddylation pathway scores as biomarkers for disease progression and prognosis in KIRC patients.

### 2.4. Relationship between Neddylation Clusters and Drug Sensitivity

**Figure 6:**
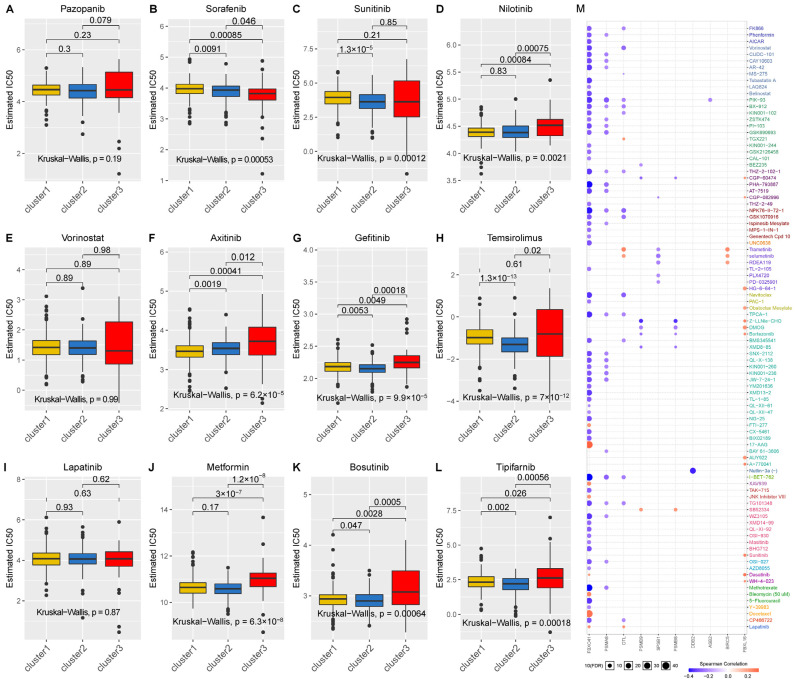
Drug sensitivity variations among different neddylation clusters. (**A**–**L**) These box plots depict the estimated IC50 values of 12 specific drugs for Cluster 1 (represented in yellow), Cluster 2 (denoted in blue), and Cluster 3 (indicated in red). The numbers positioned above the plots signify statistically significant p-values. The box plots provide a clear visual representation of the disparities in drug sensitivities amongst the neddylation clusters. (**M**) The scatter plot showcases the unique influences of GDSC drugs on the mRNA expression of neddylation class genes. The spheres present on the plot symbolize statistical significance (*p* < 0.05), whereas larger spheres demonstrate a more potent correlation. A red sphere indicates an increase in mRNA expression, while a blue sphere represents a decrease.

To investigate the relationship between the neddylation pathway and drug sensitivity, we procured data corresponding to 12 drugs from the GDSC database. We introduced a half-maximal inhibitory concentration value to compare drug sensitivity among the distinct clusters, with the principle understanding that IC50 is inversely proportional to drug sensitivity. For instance, the effectiveness of Sorafenib was denoted as C3 > C2 > C1, Sunitinib was denoted as C2 > C1, Nilotinib as C2 > C3 and C1 > C3, Axitinib as C1 > C2 > C3, Gefitinib as C2 > C1 > C3, Temsirolimus as C2 > C1 and C2 > C3, Metformin as C1 > C3 and C2 > C3, Bosutinib as C2 > C1 > C3, and Tipifarnib as C2 > C1 > C3 (Figure 6A–L). Through our analysis, Gefitinib, Temsirolimus, Bosutinib, and Tipifarnib manifested superior efficacy in patients with high neddylation scores in KIRC. Furthermore, we analyzed the response data of several classic targeted drugs pertaining to neddylation-related genes. Our findings reveal that the expression of the majority of neddylation-related genes is influenced by targeted therapeutics, commonly displaying inhibitory effects. Among these, the gene FBXO41 displays the most pronounced effect, underscoring its significance in targeted therapy for KIRC (Figure 6M).

### 2.5. The Impact of the Neddylation Score on Classical Oncogenes and Immune Infiltration

**Figure 7:**
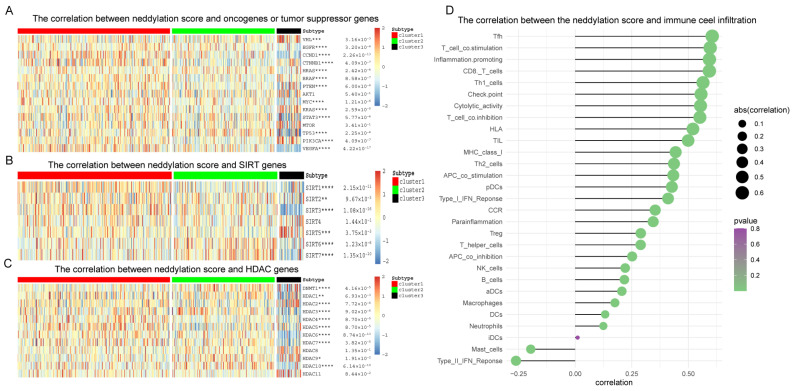
The impact of the neddylation score on classical oncogenes and immune infiltration. (**A**–**C**) The association between the neddylation score and classical cancer-associated genes, sirtuin family genes, and HDAC family genes was evaluated, with significance levels represented by * *p* < 0.05, ** *p* < 0.01, *** *p* < 0.005, and **** *p* < 0.001. (**D**) The study examined the correlation between the neddylation score and immune cell infiltration. In this figure, the sphere’s area represents the magnitude of abs (correlation), and the color indicates the corresponding *p*-value.

In our study, we observed that Cluster 3, characterized by the high expression of several proto-oncogenes including catenin beta 1 (CTNNB1), BRAF, KRAS, and PIK3CA, appears to be a critical determinant of poor prognosis in patients with KIRC (Figure 7A). This finding is particularly noteworthy given that VHL is the only tumor suppressor gene in this context. The prominence of proto-oncogenes in Cluster 3 underscores the complexity of the regulatory processes governing KIRC prognosis. Our analysis reveals that multiple genes contribute to the intricate molecular landscape of KIRC, warranting further in-depth investigation to understand their interplay and impact on patient outcomes. Similarly, in our study, we have observed that the elevated expression of the proto-oncogene SIRT1, coupled with the reduced expression of the tumor suppressor gene SIRT3, plays a significant role in contributing to the adverse prognosis observed in Cluster3 of KIRC patients (Figure 7B). 

In our study focusing on KIRC, a comprehensive analysis of DNA methyltransferase 1 (DNMT1) and HDACs was conducted. We discovered that elevated expression levels of the proto-oncogenes DNMT1, HDAC1, and HDAC2 are strongly associated with poor prognostic outcomes. This finding highlights the critical roles these genes play in KIRC pathogenesis, suggesting their potential as key factors in the disease’s progression and as targets for therapeutic intervention (Figure 7C).

A correlation analysis between the neddylation score and infiltration of various immune cells was performed. According to the results, Tfh type, T cell co-stimulation, inflammation promotion, and CD8 T cell infiltration show a positive correlation, while type II interferon (IFN)-reactive cell infiltration demonstrates a negative correlation with mast cell infiltration (Figure 7D). Moreover, a scatter plot was devised to illustrate the potent positive correlation between the neddylation score and Tfh, T cell co-stimulation, inflammation-promoting, and CD8 T cells (Figure S3A–D). The results signify a robust positive correlation between the two, which aligns with the findings presented in Figure 7D of the study. These findings suggest that both the tumor microenvironment and immune cell infiltration play pivotal roles in KIRC tumor progression, and the neddylation pathway may potentially regulate the immune response.

### 2.6. Screening for Neddylation-Related Genes with Specific Effects on KIRC

**Figure 8:**
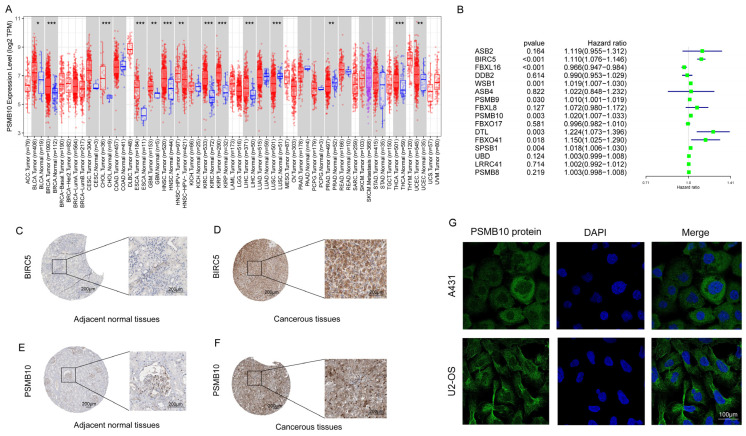
Multi-level screening of neddylation-related genes that have specific effects on the prognosis of KIRC. (**A**) The pan-cancer expression profile of PSMB10, with significance levels represented by * *p* < 0.05, ** *p* < 0.01, and *** *p* < 0.005. (**B**) Hazard ratio analysis, encompassing 95% confidence intervals and corresponding p-values. (**C**–**F**) The panel displays the immunohistochemical observations obtained from the HPA database, showcasing the protein expression of BIRC5 and PSMB10 in both KIRC tissues (T) and normal tissues (N). (**G**) The diagram depicts the immunofluorescence of PSMB10 and BIRC5 in A431 and U2-OS cell lines. In this illustration, the green fluorescence signifies the localization of PSMB10 and BIRC5 proteins, whereas the blue fluorescence represents the cell nuclei.

A pan-cancer analysis revealed that PSMB10 is significantly overexpressed in various tumor types compared to normal tissues, with notably high expression levels in KIRC (Figure 8A). Similarly, we analyzed the gene expression levels in our samples and identified an optimal cutoff value, enabling the stratification of the samples into two distinct groups: high-expression and low-expression. To elucidate the effect of these gene expression variations on patient survival, we constructed a Kaplan–Meier survival curve. Research findings demonstrated that various neddylation-related genes could either improve or deteriorate the prognosis of patients with KIRC (Figure S4A). It was revealed that elevated expression levels of ASB2, WSB1, BIRC5, ASB4, FBXO41, PSMB10, FBXL8, and SPSB1 are strongly correlated with poor patient outcomes, underscoring their potential as prognostic markers for cancer. Conversely, heightened expression levels of FBXO17, leucine-rich repeat containing 41 (LRRC41), FBXL16, UBD, and DDB2 are identified as positive prognostic indicators. Furthermore, we analyzed the hazard ratios of 16 neddylation-related genes to elucidate the relationship between their expression and the prognosis of KIRC patients. The analysis revealed that FBXL16 exhibits a protective effect, whereas BIRC5, WSB1, PSMB9, PSMB10, DTL, and SPSB1 are associated with adverse outcomes. These findings, all statistically significant (*p* < 0.05), are shown in the forest plot (Figure 8B). Leveraging the UALCAN website, we selected several representative neddylation-related genes (PSMB10, PSMB9, PSMB8, FBXO17, FBXL8, DDB2, BIRC5) and compared their protein expression discrepancies between KIRC tissues and normal tissues. The findings indicate that the neddylation-related genes protein in KIRC tissues is higher than in normal tissues (Figure S4B). 

We carried out an in-depth analysis of PSMB10, utilizing the resources available through the HPA database. To start, we contrasted the immunohistochemical staining of BIRC5 (classical oncogene) and PSMB10 in primary tumor tissues and normal tissues in cases of KIRC. The outcomes illustrated a substantial enhancement in the expression of PSMB10 proteins in KIRC tissues relative to normal tissues (Figure 8C–F). Furthermore, we scrutinized the immunofluorescence of PSMB10 in A431 and U2-OS cell lines (Figure 8G). This examination substantiated that PSMB10 is expressed predominantly in the cytoplasm. These results validate the specificity of PSMB10 expression in KIRC. We investigated the co-expression relationships of PSMB10 with various genes. Our analysis revealed that PSMB8, PSMB9, ASB2, UBD, PSMA8, BIRC5, DTL, LRRC41, WSB1, and ASB4 show a positive correlation with PSMB10 expression. In contrast, FBXL16 and SPSB1 are found to be negatively correlated with PSMB10 expression (Figure S4C). In summary, our findings highlight that PSMB10 protein displays augmented expression levels in tumor tissues compared with normal tissues and is associated with unfavorable prognostic outcomes in patients. Consequently, we plan to conduct further analysis on this gene. 

### 2.7. In Vitro Dosing Experiments Elucidate the Pivotal Role of Neddylation Modification in Determining the Phenotype of KIRC

**Figure 9:**
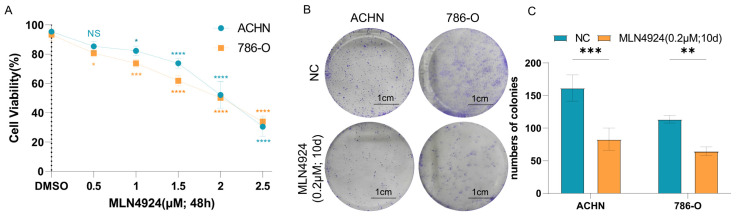
Comprehensive investigation into the effects of MLN4924-induced inhibition of neddylation modification on the KIRC phenotype. (**A**) Dose-response evaluation of MLN4924: investigation of cell proliferation in ACHN and 786-O cell lines following treatment with serial dilutions of MLN4924 (0, 0.5, 1, 1.5, 2, 2.5 μM), utilizing a CCK-8 assay to assess proliferation rates. (**B**) Colony formation assay following MLN4924 treatment: comparison of colony formation capabilities in ACHN and 786-O cell lines treated with MLN4924 (0.2 μM; 10 days) against control groups, indicating a reduction in colony-forming ability post-treatment. (**C**) Quantitative colony formation analysis: consolidated results from three independent colony formation assays highlight a significant decrease in the colony-forming capacity of ACHN and 786-O cells treated with MLN4924 (0.2 μM; 10 d) compared with controls. Statistical significance is denoted by * *p* < 0.05, ** *p* < 0.01, *** *p* < 0.001, **** *p* < 0.0001.

To assess the impact of neddylation modification inhibition on the proliferation of renal cancer cells, we administered serial dilutions of the neddylation-specific inhibitor MLN4924 (0, 0.5, 1, 1.5, 2, 2.5 μM) to ACHN and 786-O cell lines. This treatment was aimed at evaluating its capacity to decrease cell viability in vitro over a 48 h period. We observed that MLN4924 treatment for 48 h led to a significant and dose-dependent reduction in the viability of both ACHN and 786-O cell lines, with the inhibitory effect at 2 μM closely approximating the half-maximal inhibitory concentration (Figure 9A). Consequently, we selected a concentration of 2 μM for a 48 h treatment for subsequent analysis of immunofluorescence protein expression. The results demonstrated that the expression levels of NEDD8 and PSMB10 proteins are significantly diminished following MLN4924 treatment (Figure S5A–E). As mentioned previously, NEDD8 serves as a crucial marker of neddylation, and its reduced expression indicates that MLN4924 effectively inhibits neddylation modification in renal cell carcinoma. Concomitantly, the down-regulation of PSMB10 suggests its potential role as a target in the modulation of neddylation modification. 

Further, colony formation experiments show that the number of colonies in the MLN4924-treated group is significantly reduced compared with the blank control group (Figure 9B,C). Additionally, transwell invasion and migration assays indicate that treatment with 2 μM MLN4924 for 24 h significantly reduced the invasion and migration capabilities of ACHN and 786-O cell lines compared with the control group (Figure S5F–H). Scratch assays further corroborated these findings, showing diminished migration at 12 and 24 h post-treatment with 2 μM MLN4924 relative to the untreated controls (Figure S5I–K). In summary, our study confirms that inhibition of neddylation modification in renal cell carcinoma significantly impedes its proliferation, invasion, and migration. Furthermore, our data also confirm that PSMB10 may serve as a key regulator in this pathway, corroborating the insights gained from our preceding bioinformatics study on KIRC.

### 2.8. Deciphering the Impact and Underlying Mechanisms of MLN4924 Treatment on KIRC Cell Lines through RNA Sequencing Analysis

**Figure 10:**
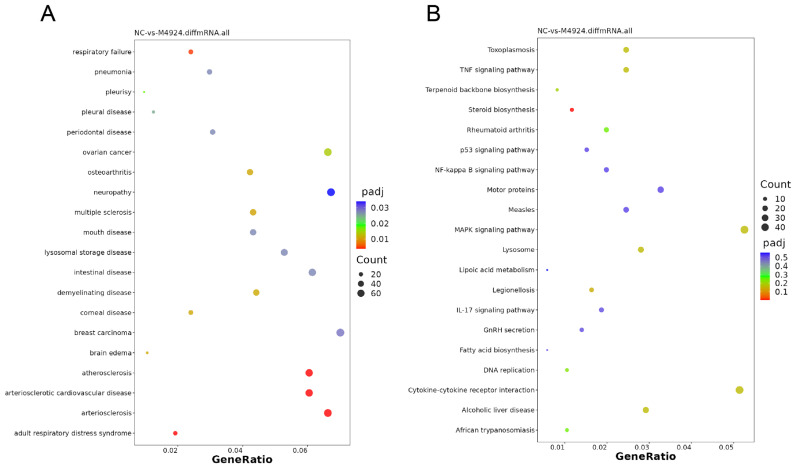
Comparative RNA sequencing analysis between MLN4924-treated and control groups. (**A**) DO enrichment bubble chart of differentially expressed genes. (**B**) KEGG enrichment bubble chart of differentially expressed genes. The size of each bubble signifies the degree of enrichment, and its position denotes the significance of the disease (pathway) affected by the gene changes.

To elucidate the mechanism of neddylation in regulating KIRC biological phenotypes, we used the 786-O cell line as a model system. This line underwent treatment with 2 μM MLN4924 for 48 h, serving as the experimental group, while a corresponding untreated set served as the control group. Each condition was replicated three times, resulting in the samples M4924-1, M4924-2, and M4924-3 (experimental) and NC-1, NC-2, and NC-3 (control), which were then subjected to transcriptomic sequencing by a specialized biotechnology firm. Quality assurance steps were meticulously carried out on the sequencing data, beginning with the assessment of total RNA integrity via agarose gel electrophoresis (Figure S6A). This was followed by an evaluation of sequencing fidelity, GC content distribution, and raw data purification, leading to the generation of clean reads for in-depth analysis. This involves the removal of reads containing sequencing adapters, exclusion of reads with an N ratio exceeding 10%, and elimination of low-quality reads, where the number of bases with a quality value (Q) ≤ 20 constitutes more than 50% of the entire read. The result is a collection of clean reads ready for analysis (Figure S6B). Comparative analysis of the sequencing outcomes revealed significant gene expression alterations between the experimental (sh-RECQ1) and negative control (NC) groups. Specifically, there was a reduction in expression levels of over 1180 genes by more than 50% (log2 ≤ −1) and an increase in 914 genes with expression levels more than doubled (log2 ≥ 1), as illustrated in the volcano plot and cluster heat map (Figure S6C,D). To interpret the biological significance of these expression changes, we conducted a Disease Ontology (DO) analysis, focusing on the gene function–disease correlation. This analysis pinpointed that the differentially expressed genes were predominantly associated with various cancer types and inflammatory diseases (Figure 10A). Furthermore, a Key Pathway Analysis via the Kyoto Encyclopedia of Genes and Genomes (KEGG) revealed substantial enrichment of these genes in critical signaling pathways implicated in KIRC malignancy, including the TNF signaling pathway, NF-kappa B signaling pathway, and the p53 signaling pathway (Figure 10B). These transcriptomic insights confirm that neddylation’s prognostic influence on KIRC is mediated through a concerted modulation of multiple signaling pathways, underscoring the complexity of its regulatory mechanisms. In particular, the observed regulatory effect of neddylation on the NF-kappa B signaling pathway reflects the feasibility of our previous studies on neddylation regulating PSMB10 expression. Because the immunoproteasome subunit (PSMB10) helps fine-tune specific intracellular pathways, including the NF-kB signaling pathway, it plays a critical role in managing immune responses, oxidative stress, and maintaining cellular proteostasis [65]. This guides us to conduct further research on the target role of PSMB10.

### 2.9. In Vitro Analysis Reveals the Targeted Therapeutic Potential of PSMB10 in KIRC

To refine the understanding of PSMB10 expression in KIRC, we conducted comparative studies using one normal renal cell line (HK2) and two KIRC cell lines (786-O and ACHN). The expression of PSMB10 was quantified using reverse transcription-polymerase chain reaction (RT-PCR). The RT-PCR data demonstrates significantly elevated levels of PSMB10 in the KIRC cell lines compared with the normal renal cells (Figure S7A). To further investigate the biological implications of PSMB10 in KIRC, we initiated a targeted reduction of its expression using small interfering RNA (siRNA) technology. Initial screening of various siRNAs led to the selection of HOMO-334 and HOMO-908, specifically for the PSMB10 knockdown in the 786-O cell line. RT-PCR analysis confirmed the superior knockdown efficiency of HOMO-908, which achieved an approximate 90% reduction in PSMB10 expression (Figure S7B). The same siRNA, HOMO-908, was then employed in the ACHN cell line, resulting in a similar decrease in PSMB10 expression (Figure S7C). The transfection efficiency in both 786-O and ACHN cell lines was validated using a negative control FAM label, as provided by the reagent supplier. This control corroborated the success of the transfection process (Figure S7D,E). Beyond transcriptional analysis, we also assessed PSMB10 protein expression post-siRNA (HOMO-908) knockdown in both ACHN and 786-O cell lines using immunofluorescence (Figure S7F–I). These investigations confirmed the reliability of siRNA (HOMO-908) in effectively targeting the PSMB10 gene. Consequently, HOMO-908 was selected for subsequent knockdown experiments within this study.

**Figure 11:**
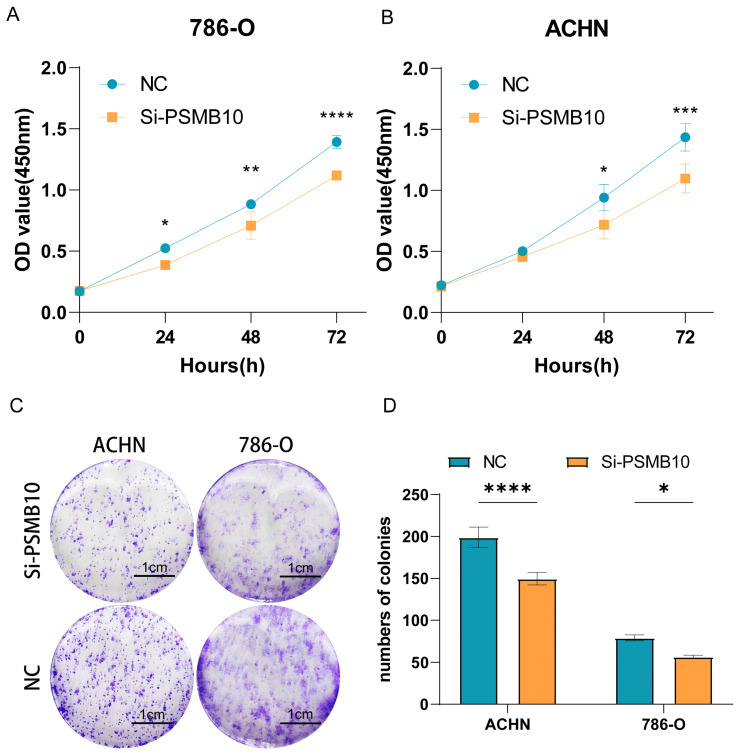
Comprehensive analysis of PSMB10 gene expression and functional impact on KIRC cell lines. (**A**,**B**) Cell proliferation assessment using CCK-8 assay in ACHN and 786-O cell lines post Si-PSMB10 and NC (negative control) transfection. (**C**) Colony formation assay in ACHN and 786-O cell lines following transfection with Si-PSMB10 and NC. (**D**) Quantitative analysis of three independent colony formation experiments. Impact of PSMB10 knockdown on cell migration and invasion in ACHN and 786-O cell lines. * *p* < 0.05, ** *p* < 0.01, *** *p* < 0.001, **** *p* < 0.0001.

A CCK-8 assay was utilized to evaluate the effect of PSMB10 knockdown on cell proliferation in 786-O and ACHN cell lines. The assay revealed a significant decrease in proliferation in these cell lines following PSMB10 knockdown. Notably, in the 786-O knockdown group, significant deviations from the control group were observed starting from 24 h, while in the ACHN cells, these differences became apparent after 48 h (Figure 11A,B). To further elucidate the impact of PSMB10 on the proliferative behavior of KIRC cells, clonogenic assays were conducted. The results from these assays indicated a pronounced reduction in both the quantity and size of the colonies formed by the 786-O and ACHN cells post-PSMB10 gene knockdown (Figure 11C,D). The scratch assay results demonstrated that the migratory capacity of the 786-O and ACHN cell lines was markedly impaired at 12 and 24 h following PSMB10 knockdown (Figure S7J–L). This observation was further corroborated by the transwell cell migration assay, which yielded consistent findings. Additionally, the results of the transwell cell invasion assay indicated a substantial decrease in the invasive potential of these cell lines post-PSMB10 knockdown (Figure S7M–O). Together, these experimental outcomes provide robust evidence supporting the pivotal role of PSMB10 in regulating the migration and invasion characteristics of KIRC cells.

## 3. Discussion

The advancement of structural and molecular biology research has progressively unraveled the potential mechanism underlying the neddylation cascade, along with its pivotal role in tumor biology [66,67,68]. The neddylation pathway exhibits substantial influence over the tumor microenvironment (TME) [69]. It is widely acknowledged that the TME, an intricate network comprising both cells and the extracellular matrix, provides an environment conducive to tumor cell proliferation [70]. The TME is known to have a decisive role in the regulation of tumor growth, metastasis, and response to therapy [71]. Tumors are comprised not only of malignant cells but also a variety of normal cells, such as fibroblasts, endothelial cells, lymphocytes, and macrophages [70,72,73]. The emerging paradigm suggests that therapeutic targeting of the TME, through modulation of the neddylation pathway, may represent a novel strategy in cancer treatment [74]. 

Renal cell carcinoma (RCC) is characterized by significant heterogeneity, metastatic potential, and immune reactivity [75]. Among RCC subtypes, KIRC is the most prevalent [76,77]. For early-stage KIRC, treatment options are largely limited to surgical interventions. While these interventions can be effective, postoperative recurrence is observed in 20–40% of patients [78]. Traditional surgical and medical treatments often prove infeasible for patients with advanced stages of the disease [79]. Nevertheless, this gap is being increasingly addressed by targeted therapies. The efficacy of targeted therapy lies in its ability to tailor treatment to individual gene mutation sites, a strategy that is now extensively implemented in clinical practice [80]. Despite the escalating incidence of KIRC, the synergistic effects of combining molecular targeted drugs with immunotherapy can enhance treatment effectiveness and ameliorate patient prognosis. Additionally, the burgeoning progress in the field of neddylation-KIRC research has begun to draw significant attention [81].

Our study reveals a substantial presence of CNVs and SNVs in neddylation-related genes. Notably, these genes exhibit a more pronounced expression in KIRC compared to other tumor types. At the cellular pathway level, neddylation-related genes demonstrate a significant regulatory impact on crucial processes such as cell apoptosis and cell cycle regulation. We get further in-depth investigation to elucidate their role in cancer biology and their potential as therapeutic targets.

DNA methylation, a critical epigenetic modification crucial for gene regulation in the mammalian genome, typically involves the addition of a methyl group to the cytosine base in DNA. This process, extensively documented in the scientific literature [82,83], plays a pivotal role in either suppressing or promoting gene expression. Gene suppression is achieved mainly by recruiting regulatory proteins that alter chromatin structure into a repressive state or by preventing transcription factors from binding to DNA, thus inhibiting gene transcription [84]. In cancer biology, alterations in DNA methylation patterns, such as hypermethylation or hypomethylation, lead to dysregulated gene expression, contributing to tumorigenesis. For instance, hypermethylation in promoter regions of tumor suppressor genes results in their silencing, while hypomethylation can activate oncogenes. These epigenetic changes are closely linked to various clinicopathological features in cancers, affecting tumor progression, metastasis, and patient prognosis, ultimately impacting survival [85]. Our research reveals that neddylation-related gene DNA methylation alterations are widespread in cancerous tissues. These prevalent modifications affect the pathophysiology of KIRC, suggesting that targeting neddylation-related methylation patterns could offer novel therapeutic strategies, potentially improving survival outcomes for KIRC patients. 

Meanwhile, our heatmap displayed marked disparities in the expression of neddylation-related genes between tumor and healthy tissues. This observation led us to stratify KIRC samples into three clusters—low, normal, and high—based on the neddylation score for subsequent cluster analysis. Our study’s analysis of the survival curves suggests an imbalance characterized by either excessively high or low expression levels of neddylation-related genes appears detrimental to patient prognosis. Furthermore, we have identified that the prognostic influence exerted by these genes may be mediated through various clinical parameters, including tumor grade (T), cancer stage, and patient status (Fustat). This intricate interplay underscores the nuanced role of neddylation-related genes in the pathophysiology of the disease and highlights their potential as biomarkers or therapeutic targets in the management of cancer patients.

In regards to targeted therapy it has emerged as a transformative modality to enhance the prognosis of advanced KIRC patients and ameliorate the adverse effects triggered by other treatment modalities [86,87]. The quest for the most suitable targeted therapeutic drug to address the heterogeneity inherent in KIRC has been a focal point of our research. We probed the variance in sensitivity to frequently utilized targeted drugs for KIRC treatment across the three neddylation-related gene clusters. The insights gathered underscore the potential for tailoring treatment plans based on individual neddylation score profiles. This classification based on neddylation clusters and drug sensitivity is not just a theoretical construct; it has direct, actionable implications for the treatment of KIRC. By understanding the differential responses of neddylation-related genes to various drugs, we can guide more personalized and effective treatment strategies for KIRC patients. For instance, patients with high neddylation scores might benefit more from Gefitinib, Temsirolimus, Bosutinib, and Tipifarnib. Similarly, targeting FBXO41 could be a promising therapeutic strategy. Our findings thus pave the way for more tailored and potentially more effective treatments for KIRC, moving us closer to personalized medicine in oncology.

We also studied the protein translation modification of neddylation-related genes. The protein translation modification refers to the changes in the amino acid side chains of certain proteins after biosynthesis. These modifications carried out through the functional groups’ covalent addition can influence proteolysis cleavage of the subunit or degradation of the entire protein, significantly impacting standard cellular biology and disease mechanisms [88]. Acetylation and deacetylation of histones, as primary protein translational modifications, are crucial processes in chromatin modification and are considered pivotal determinants for the alternation between chromatin’s open and closed states [89]. Furthermore, protein acetylation modification can modulate its activity, localization, specificity, and interaction modality, thereby influencing the protein’s function and signal transduction pathways [90,91]. The exploration of these modifications bears considerable biological and clinical relevance in domains such as cancer and neurodegenerative diseases. Our research denotes a correlation between most classical oncogenes, SIRTs and HDACs, with the neddylation pathway.

We identified that CTNNB1, BRAF, KRAS, and PIK3CA are proto-oncogenes exhibiting high expression in Cluster 3 of KIRC patients. CTNNB1, a crucial component of the Wnt signaling pathway, is implicated in driving cell proliferation and inhibiting apoptosis upon overexpression [92,93]. BRAF, integral to the MAPK/ERK signaling pathway, plays a significant role in cancer progression [94], and its mutations are associated with enhanced cell growth and survival [95]. KRAS, a well-recognized oncogene across various cancers, activates key pathways such as MAPK and PI3K, leading to increased cell proliferation, survival, and migration [96,97]. PIK3CA, functioning within the PI3K/AKT/mTOR pathway, is known to contribute to tumorigenesis and resistance to apoptosis when overexpressed, underlining its crucial role in cell growth and survival [98]. Our findings suggest that the high expression of these proto-oncogenes in Cluster 3 may be a primary contributor to the poor prognosis observed in patients with clear cell renal cell carcinoma, indicating their potential as targets for therapeutic intervention and prognostic markers in this disease.

SIRTs assume significant roles in fundamental physiological procedures such as cellular metabolism, stress response, and circadian clock regulation. By extracting acetyl groups from a range of proteins, sirtuins can influence various cellular processes, including apoptosis, metabolism, development, and aging [99]. SIRT1, commonly recognized as a proto-oncogene in various cancers, is known for its involvement in promoting cell survival and proliferation [100]. Its overexpression has been consistently linked to tumorigenesis across several cancer types [101]. On the other hand, SIRT3 generally functions as a tumor suppressor, primarily by regulating mitochondrial function and oxidative stress [102]. In the context of KIRC, SIRT3 appears to maintain this role, though the specific effects are likely influenced by the cancer’s unique genetic and epigenetic makeup. These findings highlight the crucial roles of SIRT1 and SIRT3 in the pathophysiology of KIRC, underscoring their potential as biomarkers for prognosis and as targets for therapeutic intervention. In this study, we have meticulously analyzed the expression patterns of key genes in KIRC, particularly focusing on Cluster3 patients. Our findings indicate that an upregulated expression of the proto-oncogene SIRT1, in conjunction with a down-regulated expression of the tumor suppressor gene SIRT3, substantially contributes to the unfavorable prognosis observed in this patient cohort. The interplay between SIRT1 and SIRT3 appears to be a critical factor in the disease progression of KIRC, suggesting that these genes may serve as potential biomarkers for prognosis or as targets for therapeutic intervention in this subset of patients.

Results from studies targeting HDAC showed a correlation between increased expression of proto-oncogenes DNMT1, HDAC1, and HDAC2 and poor prognostic outcomes in KIRC patients. Specifically, DNMT1, typically regarded as a proto-oncogene in various cancers, including KIRC, plays a critical role in DNA methylation [103]. This process is key to the silencing of tumor suppressor genes and the activation of oncogenic pathways. HDAC1, often functioning as a proto-oncogene, is implicated in chromatin remodeling [104,105]. This activity is associated with the down-regulation of tumor suppressor genes and has been linked to the pathogenesis of various cancers, including potentially KIRC. Similarly, HDAC2 is generally categorized as a proto-oncogene due to its involvement in epigenetic modulation, contributing to oncogenesis through the repression of tumor suppressor genes [106]. These insights into the roles of DNMT1, HDAC1, and HDAC2 not only enhance our understanding of KIRC pathophysiology but also open up potential avenues for targeted therapeutic strategies.

Tumor-associated inflammation has been implicated as a predictor of adverse prognosis and an instigator of diverse oncogenic phenotypes [107,108]. Our research probed into the interaction between immune cells and neddylation-related genes. We uncovered a positive correlation with the infiltration of T follicular helper (Tfh) cells, T cell co-stimulation, inflammation promotion, and CD8 T cell infiltration. Conversely, a negative correlation was observed with the infiltration of mast cells and type II IFN response cells, insinuating certain constraints of the neddylation pathway in modulating the immune activity of these cells. These novel insights pave the way for new therapeutic targets and strategies in the realm of KIRC immunotherapy.

After understanding the characteristics of the role of neddylation-related genes in KIRC, we began to comprehensively search for specific neddylation-related genes that may significantly affect the treatment of KIRC, for which we conducted extensive data analysis. Our findings reveal that PSMB10 is not only overexpressed in KIRC tumor tissues but also has a profound negative impact on patient prognosis. This overexpression of PSMB10 in the tumor milieu might be contributing to the altered cellular processes in KIRC, suggesting its potential as a therapeutic target. The role of PSMB10 in the neddylation pathway, which is crucial for protein homeostasis and cell cycle regulation, further underscores its significance in KIRC pathogenesis and as a promising avenue for targeted therapy.

To enhance the understanding of neddylation modification’s impact on KIRC and to elucidate the role of PSMB10 within this context, comprehensive in vitro experimental analyses were conducted. In our study, we specifically targeted the inhibition of neddylation modification in KIRC cells using the small molecule inhibitor MLN4924. The subsequent observations revealed a notable attenuation in the proliferation, migration, and invasion capabilities of KIRC cell lines, enhancing our understanding of neddylation modification’s contribution to KIRC’s adverse prognosis. Concurrently, a correlation was observed between the PSMB10 expression and neddylation modification levels. Integrating these observations with RNA sequencing data revealed that genes affected by diminished neddylation modification are predominantly involved in critical signaling pathways related to KIRC pathogenesis, including the TNF, NF-kappa B, and p53 signaling pathways. This provides partial clarification of the mechanisms through which neddylation modification influences KIRC phenotypes. Importantly, the interaction between neddylation modification and the NF-kappa B signaling pathway corroborates our prior research on its regulatory effect on PSMB10 expression. Considering the regulation of immunoproteasome is key to adjusting specific intracellular pathways, including the NF-kB signaling pathway, thus revealing a potential mechanism through which neddylation modulates KIRC malignancy and prognosis [65]. Meanwhile, our research has elucidated that PSMB10, a gene fundamentally involved in the ubiquitin-proteasome system, is markedly overexpressed in KIRC tumor tissues. This overexpression correlates with a notably detrimental effect on patient prognosis. The heightened presence of PSMB10 within the tumor environment appears to be a significant factor in modifying cellular processes associated with KIRC, thereby highlighting its potential as a strategic target in treatment modalities. To substantiate our findings, we executed a series of cell-based assays focusing on PSMB10. These experiments revealed that PSMB10 notably enhances the proliferation, migration, and invasion of KIRC cells. This evidence firmly establishes the substantial therapeutic implications of targeting PSMB10, reinforcing its critical role as a potential intervention point in KIRC treatment strategies. 

## 4. Materials and Methods

### 4.1. Date Extraction Processing

Leveraging the resources of the GSEA website (http://www.gsea-msigdb.org/gsea/index.jsp, accessed on 1 December 2022), we selected a set of 17 genes critically associated with neddylation-related pathways for subsequent analysis. TCGA database, a rich repository of genetic and clinical cancer-related data (https://tcga-data.nci.nih.gov/tcga/, accessed on 1 December 2022) [109], served as our foundational database. We exploited data from the TCGA database to conduct an extensive analysis of CNVs, SNVs, and gene expression levels across 33 diverse cancer types, deploying Perl and RStudio as our computational tools. The resultant data were visualized employing the Toolbox for Biologists. For an in-depth analysis, we amalgamated these data with clinical pathological data sourced from the TCGA database, resulting in hierarchical clustering performed using the GSCALite website. The threshold for statistical significance was established at a *p*-value less than 0.05.

### 4.2. Methylation

We utilized the GSCALite website (https://guolab.wchscu.cn/GSCA/#/, accessed on 1 December 2022) to probe the association between the methylation status of neddylation-related genes and a spectrum of 14 distinct cancer types. Moreover, we scrutinized the interrelationships between methylation of neddylation-related genes, mRNA expression levels across various cancer types, and corresponding cancer survival rates. The threshold for determining statistical significance was set at a *p*-value less than 0.05.

### 4.3. Cluster Analysis

In our study on differentially expressed genes, we employed the “gplots” package in RStudio for initial data visualization, followed by in-depth cluster analysis using heatmaps generated via the “pheatmap” package. Specifically, we configured the parameters to properly align tumor and normal sample data, facilitating direct comparisons. We established a gene expression threshold to categorize the genes according to their expression levels and predefined the number of sample clusters as three. Use the neddylation score calculated by GSVA to verify the difference. GSVA, a non-parametric and unsupervised approach, is widely adopted in bioinformatics for its efficacy in assessing pathway activity changes across sample populations within gene expression datasets [110]. This method transforms gene expression matrices from individual samples into gene set expression matrices, thus simplifying the comparison of metabolic pathway enrichments across different samples [110]. We constructed an expression matrix for neddylation-related genes, identifying those with significant differential expression. We calculated the standard deviation for each gene, selecting those that exhibited substantial differences between tumor and normal samples, and categorized them as either overexpressed or underexpressed relative to normal samples. The derived state matrix was then hierarchically clustered using the ward.D method.

Following the GSVA, we assigned scores to neddylation-related genes and stratified the samples into three distinct clusters: Cluster 1 with a medium score, Cluster 2 with a high score, and Cluster 3 with a low score. To confirm the validity of these clusters, we employed the “ggpubr” package in RStudio to generate violin plots, which detailed the kernel density distributions among the clusters, offering a visual contrast of their gene expression profiles. Moreover, we conducted a survival analysis to assess the patient’s prognosis, presenting the results through Kaplan–Meier curves. The integrity of our cluster analysis was further reinforced by creating heatmaps with the “pheatmap” function to display the levels of gene expression alongside associated clinicopathological features. We conducted all statistical analyses with a stringent significance threshold, setting the *p*-value at less than 0.05 to ensure a robust evaluation of our findings.

### 4.4. Drug Sensitivity

The genomics of drug sensitivity in cancer (GDSC) database (https://www.cancerrxgene.org/, accessed on 1 December 2022) is an open-source and freely accessible repository that houses a vast spectrum of information pertinent to cancer research [111]. Following the computation of half-maximal inhibitory concentration (IC50) values for samples in the three clusters, we employed the pRRophetic algorithm incorporated within the “pRRophetic” package. Box plots were generated utilizing the “ggplot2” and “cowplot” packages. Additionally, we selected ten genes related to neddylation and scrutinized their correlation with drug sensitivity data retrieved from the GDSC database. A threshold for statistical significance was set at a *p*-value less than 0.05.

### 4.5. Classic Cancer-Related Genes and Histone Modifications

To elucidate the roles of classical oncogenes, sirtuins (SIRTs), and histone deacetylases (HDACs), we employed packages such as “string”, “gplots”, “grid”, and “pheatmap” to generate a heatmap. This permitted us to visually represent diverse expression patterns of canonical oncogenes across the three distinct clusters. Our findings were deemed statistically significant at a *p*-value threshold of less than 0.05.

### 4.6. Immune Cell Infiltration

Immune cell infiltration serves as a pivotal biological process within the tumor microenvironment. In our study, we leveraged GSVA in conjunction with gene expression data extracted from the TCGA database. Our methodology facilitated the generation of a heatmap that depicted the correlations among various immune cell types. These results offer significant references and guidance for comprehending the patterns and mechanisms of immune cell infiltration within the tumor microenvironment. Furthermore, these findings provide a theoretical foundation for the development of novel immunotherapeutic strategies. We graphically represented the correlation between 29 immune cells, regulatory factors, and neddylation-related gene classes using a histogram, with an emphasis on the four most strongly correlated immune cell types. For data manipulation, analysis, and visualization in the forms of heatmaps and scatter plots, we utilized various R packages, including “data.table”, “dplyr”, “tidyverse”, “ggplot2”, and “ggstatsplot”.

### 4.7. Screening Specific Genes

To screen for the specific neddylation-related gene, we capitalized on the UALCAN database (http://ualcan.path.uab.edu/index.html, accessed on 1 December 2022), comparing protein expression results between primary KIRC tumors and normal tissues. Similarly, the HPA (https://www.proteinatlas.org, accessed on 1 December 2022), an openly accessible database, provides data on the expression and localization of human proteins within tissues and cells under both physiological and pathological conditions. Through the application of immunohistochemistry, we were able to illustrate the expression levels of BRIC5 and PSMB10 within both KIRC tumors and normal tissues. Furthermore, using immunofluorescence, we ascertained the localization of PSMB10 within A431 and U2-OS cell lines.

### 4.8. Cell Culture

The human renal proximal tubule epithelial cell line HK-2, along with the RCC cell lines 786-O and ACHN, were acquired from the Shanghai Cell Bank of the Chinese Academy of Sciences in Shanghai, China. The HK-2 cell line was cultured in Dulbecco’s modified Eagle medium (DMEM) sourced from Gibco, Life Technologies, Grand Island, NY, USA. The 786-O line was maintained in an RPMI-1640 medium provided by KeyGEN BioTECH, Nanjing, China, while the ACHN cells were cultured in a minimum essential medium (MEM) from Biosharp, Hefei, China. Each culture medium was supplemented with 10% fetal bovine serum (FBS), also obtained from Gibco, Life Technologies, Grand Island, NY, USA. The compound MLN4924 utilized in the dosing experiments was purchased from MedChemExpress (Shanghai, China) and was dissolved in dimethyl sulfoxide (DMSO, Sigma, Darmstadt, Germany) and stored at −20 °C. All cell cultures were nurtured in a controlled environment, characterized by a humidified atmosphere containing 5% CO_2_ and a constant temperature of 37 °C.

### 4.9. RNA Sequencing

For our study, we utilized the 786-O renal carcinoma cell line as a model system. Cells were treated with 2 μM of the neddylation inhibitor MLN4924 for 48 h, which constituted the experimental group. Correspondingly, cells without treatment were used as the control group. Both conditions were replicated three times, resulting in six samples labeled as M4924-1, M4924-2, and M4924-3 (experimental) and NC-1, NC-2, and NC-3 (control). These samples were then sent for transcriptomic sequencing to APExBIO Technology LLC (Shanghai, China). 

The quality of the sequencing data was rigorously assessed, with results summarized in Table S1. This table includes the statistical analysis of the percentage of bases with Phred quality scores above 20 and 30 (Q20, Q30), as well as the GC content of the sequenced DNA. Alignment to the reference genome was performed using HISAT2, an alignment tool that utilizes an enhanced version of the Burrows–Wheeler Transform (BWT) algorithm. The details of this alignment process are provided in Table S2. Subsequent to alignment, the StringTie tool was employed to quantify gene expression levels by counting the reads covering each gene from start to end. Each sample’s gene expression was quantified independently using StringTie, and the data were then merged to construct an expression matrix for all samples, detailed in Table S3. For differential expression analysis, we employed DESeq2 for conditions with replicated samples and EdgeR for conditions without replication. Input data for these analyses were read count values obtained from the StringTie quantification process. The DESeq2 analysis involved three major steps: (1) normalization of raw read counts to correct for variations in sequencing depth; (2) utilization of a statistical model to calculate p-values for hypothesis testing; and (3) adjustment for multiple hypothesis testing to determine the False Discovery Rate (FDR), with adjusted p-values (padj) reported. Genes were selected as significantly differentially expressed based on criteria of a log2 (Fold Change) greater than 1 and an adjusted *p*-value (padj) less than 0.05, as detailed in Table S4.

### 4.10. Cell Transfection (Six-Well Plate as an Example)

Cells were cultured in appropriate growth media (MEM, RPMI-1640, or DMEM) until they reached 60–80% confluence. This confluence level was carefully monitored to ensure optimal transfection efficiency. For each transfection, the siRNA-lipid complex was prepared in a two-step process. Initially, 7.5 μL of siRNA (specific for PSMB10) was diluted in 200 μL of corresponding serum-free medium. This mixture was incubated at room temperature for 15 min to allow siRNA to dissolve adequately. Simultaneously, 5 μL of GP-transfect-mate was diluted in another 200 μL of the same serum-free medium and incubated for 15 min at room temperature. Following these incubation periods, the GP-transfect-mate was gently combined with the siRNA solution. This combined mixture was then incubated for an additional 20 min to allow complex formation.

Prior to transfection, the existing medium was aspirated from the cells, which were subsequently washed once with phosphate-buffered saline (PBS, Beyotime, Shanghai, China). Each well was replenished with 1.6 mL of fresh serum-free medium. Then, 400 μL of the siRNA-lipid complex was added to each well. The plates were gently agitated to ensure uniform distribution of the transfection mixture. Cells were then incubated at 37 °C in a 5% CO_2_ atmosphere for 4–6 h to facilitate siRNA uptake. In cases where the transfected sequence is the negative control FAM (fluorescein amidite) label, the effectiveness of the transfection can be observed using a fluorescence inverted microscope (Leica, Germany). The final concentration of siRNA used for each transfection was 75 nM. After the incubation period, the transfection medium was replaced with 2 mL of complete medium containing serum. This step was crucial to resume normal cell growth and metabolic activities post-transfection.

The GP-transfect-mate and siRNA sequences targeting PSMB10 were specifically designed and synthesized by Shanghai GenePharma. The sequences used included two interfering sequences (PSMB10-Homo-334 and PSMB10-Homo-908) and one negative control sequence labeled as negative control FAM, with their detailed sequences provided in Table 1.

### 4.11. Quantitative Real-Time PCR

Total RNA was extracted from cell samples using TRIzol reagent (ABclonal, Woburn, MA, USA), adhering to the manufacturer’s guidelines. The quality and concentration of the RNA were ascertained using a microplate reader (Hybaid, Franklin, MA, USA). For reverse transcription, the cDNA synthesis was conducted using the TransScript^®^ All-in-One First-Strand cDNA Synthesis SuperMix for qPCR Kit (TRAN, Beijing, China), following the manufacturer’s instructions. The reaction mixture for each sample consisted of 2 μL of Total RNA/mRNA, 4 μL of 5 × TransScript^®^ All-in-One SuperMix for qPCR, 1 μL of gDNA Remover, and 13 μL of RNase-free water, culminating in a total volume of 20 μL. The reaction conditions were set at 25 °C for 5 min, 50 °C for 30 min, and a final inactivation step at 85 °C for 5 min.

Quantitative real-time PCR (qRT-PCR) was subsequently performed to evaluate gene expression levels using a PerfectStart^®^ Visual Green qPCR SuperMix Kit (TRAN, Beijing, China). Each reaction mixture contained 4 μL of cDNA, 0.4 μL each of Forward and Reverse Primers, 10 μL of 2×PerfectStart^®^ Visual Green qPCR SuperMix, and 5.2 μL of Nuclease-free water, resulting in a total volume of 20 μL. The thermal cycling conditions included an initial denaturation at 95 °C for 5 min, followed by 40 cycles at 95 °C for 10 s, 60 °C for 30 s, and 72 °C for 30 s. Glyceraldehyde-3-phosphate dehydrogenase (GAPDH) was employed as an internal control for normalizing gene expression. The primers for the target gene (PSMB10) and GAPDH were designed and synthesized by Tsingke Biotechnology Company (Beijing, China), with their detailed sequences provided in Table 1. Data analysis was performed using the 2^−ΔΔCT^ method.

### 4.12. Immunofluorescence

In the immunofluorescence protocol for 786-O and ACHN cell lines, cells are first fixed with 4% formaldehyde, followed by permeabilization using 0.5% Triton X-100 for 15 min and washing with phosphate-buffered saline (PBS). They are then blocked with 1% Bovine Serum Albumin (BSA) for 30 min at room temperature. Overnight incubation at 4 °C in a humidified chamber is conducted using a diluted PSMB10 primary antibody (Catalog No: HA500393, HUABIO, Hangzhou, China). The subsequent day involves exposure to a diluted fluorescently labeled secondary antibody (catalog No: AS073, ABclonal, Woburn, MA, USA) for one hour in darkness to prevent photobleaching, followed by a 5 min DAPI stain (catalog No: SI111, SEVENBIO, Shanghai, China) for nuclear visualization. The procedure is finalized with fluorescence microscopy imaging.

### 4.13. CCK-8 Cell Proliferation Assay

The process of cell seeding and transfection was conducted following established protocols. The transfected renal cancer cells were treated with 0.25% trypsin (sourced from HyClone, Logan, UT, USA), counted, and then 3000 of these cells were seeded into each well of a 96-well plate. Each experimental group consisted of three replicate wells. A volume of 100 μL of complete medium was added to each well, while the surrounding area was filled with PBS to minimize water evaporation. The plates were incubated at 37 °C in a 5% CO_2_ environment for 24, 48, and 72 h. At these predetermined time intervals, the existing medium was discarded, and the wells were refilled with 100 μL of serum-free medium mixed with 10 μL of CCK-8 solution (procured from MCE, Monmouth Junction, NJ, USA). It was essential to protect the plates from light exposure to prevent degradation of the CCK-8 reagent. Afterward, the plates were placed back in the incubator for additional periods of 0.5, 1, and 2 h. Cell viability was subsequently measured using a microplate reader (Hybaid Corporation, Franklin, MA, USA) at an absorbance of 450 nm.

### 4.14. Colony Formation

The cells were distributed into a 6-well plate, with each well containing approximately 800 cells. They were then incubated at 37 °C in a 5% CO_2_ atmosphere for around 10 days to encourage colony formation. Post-incubation, cell colonies were meticulously inspected under a light microscope (Leica, Wetzlar, Germany). To remove any non-adherent cells and debris, the cells were carefully washed twice with phosphate-buffered saline (PBS). Following this, they were fixed in 4% paraformaldehyde (PFA) for 15 min at room temperature, with the PFA being sourced from Beyotime Company, Shanghai, China. After fixation, the cells were stained with 0.1% crystal violet (Solarbio, Beijing, China) for 20 min to facilitate colony observation. The excess stain was then gently washed off with distilled water. Finally, the plates were air-dried at room temperature, and colonies in each well were quantified under standard white light.

### 4.15. Transwell Invasion Experiment

In a biosafety cabinet, a transwell chamber (HTS Transwell-24 units w/0.8 μm pore polyester membrane and 6.5 mm inserts, Corning, Corning, NY, USA) was prepared by evenly distributing 30 μL of Matrigel^®^ Growth Factor Reduced (GFR) Basement Membrane Matrix, LDEV-free (Corning, USA) on the upper chamber’s underside; this setup was then air-dried for 2 h and incubated overnight at 37 °C with 5% CO_2_. Transfected renal cancer cells were trypsinized, washed with PBS and serum-free medium, and resuspended in serum-free medium to a concentration of 2 × 10^5^ cells/mL. To the bottom of a 24-well plate, 600 μL of medium with 20% serum was added, followed by 200 μL of the cell suspension to the upper chamber, and the plate was incubated for another 24 h. Post-incubation, the transwell chambers were carefully removed with tweezers, the upper chamber’s liquid was aspirated, and the chambers were transferred to wells containing 800 μL of 4% PFA for a 30 min fixation at room temperature. Following fixation, the chambers were moved to wells containing 800 μL of crystal violet for 30 min staining at room temperature. After several gentle PBS washes, the non-invading cells on the upper membrane surface were wiped off with a wet cotton swab. Using brightfield illumination, count the cells that have invaded through the membrane in three randomly selected fields under the microscope.

### 4.16. Cell Migration Experiment

Cell culture, plate inoculation, and transfection were conducted as per established methods. Transfected renal cancer cells were trypsinized, washed with PBS and serum-free medium, and resuspended in serum-free medium to a density of 2 × 10^5^ cells/mL. Subsequently, 600 μL of medium with 20% serum was added to the bottom of a 24-well plate, with 200 μL of the cell suspension placed in the upper chamber, which was then incubated for 24 h. Post-incubation, the upper chamber was removed, the liquid aspirated, and the chamber transferred to a well containing 800 μL of 4% PFA for a 30 min fixation at room temperature. The fixed chambers were then stained with 800 μL of crystal violet for 30 min at room temperature. After rinsing with PBS, the non-migration cells on the upper membrane were wiped off with a wet cotton swab. Count the migrated cells in three randomly selected microscope fields using brightfield illumination and statistically analyze the results.

### 4.17. Wound Healing Assay

Transfected ACHN and 786-O cells were seeded into 6-well plates. A sterile 200 μL pipette tip was used to create scratches on the cell monolayer. Following this, the cells were washed three times with PBS to remove any detached cells. The progress of cell migration into the scratched area was then monitored using a fluorescence-inverted microscope (Leica, Germany). Observations were made at the initial point (0 h) and subsequently at 12 and 24 h to evaluate the wound healing response.

### 4.18. Statistical Analysis

Statistical analysis was conducted utilizing GraphPad Prism version 9.5 software. Data, representing values from at least three independent experiments, are reported as the mean ± standard deviation. A *p*-value of less than 0.05 was established as the threshold for statistical significance.

## 5. Conclusions

In summary, our study integrates bioinformatics analysis, in vitro experiments, and RNA sequencing to elucidate the significant role of neddylation modification in influencing the prognosis of KIRC, with a specific focus on PSMB10 as a regulatory target. These findings underscore the potential of personalized, targeted therapies that utilize differential expression of neddylation-related genes, offering a promising strategy to enhance patient outcomes.

## Figures and Tables

**Table 1:** Oligonucleotides used in research.

Oligonucleotides		Nucleotide Sequence
siRNA	PSMB10-Homo-334	Sense: GCUGCGAGAAGAUCCACUUTT
		Antisense: AAGUGGAUCUUCUCGCAGCTT
	PSMB10-Homo-908	Sense: GGAGCUAGUGGAGGAAACUTT
		Antisense: AGUUUCCUCCACUAGCUCCTT
	Negative control FAM	Sense: UUCUCCGAACGUGUCACGUTT
		Antisense: ACGUGACACGUUCGGAGAATT
Primer	GAPDH	Forward 5′-TGAAGGGTGGAGCCAAAAG-3′
		Reverse 5′-AGTCTTCTGGGTGGCAGTGAT-3′
	PSMB10	Forward 5′-GGCAATGTGGACGCATGTG-3′
		Reverse 5′-CTCCACTAGCTCCAGGGTTAGT-3′

## Data Availability

The original contributions presented in the study are included in the article. Further inquiries can be directed to the corresponding authors.

## Supplementary Materials

The following supporting information can be downloaded at: https://www.mdpi.com/article/10.3390/ph17050635/s1, Figures S1–S7 supplement Figure 3, Figure 5, Figure 7, Figure 8, Figure 9, Figure 10 and Figure 11 in the article, providing additional insights into the research findings. Figure S1: Widespread genetic mutations in neddylation-related genes; Figure S2: Comprehensive cluster analysis conducted predicated on neddylation scores; Figure S3: The impact of the neddylation score on immune infiltration; Figure S4: Multi-level screening of neddylation-related genes that have specific effects on the prognosis of KIRC; Figure S5: Comprehensive investigation into the effects of MLN4924-induced inhibition of neddylation modification on the KIRC phenotype; Figure S6: Comparative RNA sequencing analysis between MLN4924-treated and control groups; Figure S7: Comprehensive analysis of PSMB10 gene expression and functional impact on KIRC cell lines. Tables S1–S4 complement the RNA-seq portion of the article, offering detailed supplementary data. Table S1: Summary of sequencing data quality; Table S2: reference sequence alignment results; Table S3: Quantification of gene expression; Table S4: Differential analysis.

## Glossary

ASB	Ankyrin repeat and SOCS box protein
BCA3	Breast cancer-associated protein 3
BIRC5	Baculoviral IAP repeat containing 5
CCK-8	Cell Counting Kit-8
CNV	Copy number variation
CRLs	Cullin-RING ligases
CSN	COP9 signalosome
CTNNB1	Catenin beta 1
DDB2	DNA damage-binding protein 2
DMEM	Dulbecco’s modified Eagle medium
DNMT1	DNA methyltransferase 1
DTL	Denticleless E3 ubiquitin protein ligase homolog
EMT	Epithelial-mesenchymal transition
FBXL	F-box and leucine-rich repeat protein
FBXO	F-box protein
FBS	Fetal bovine serum
GAPDH	Glyceraldehyde-3-phosphate dehydrogenase
GDSC	Genomics of drug sensitivity in cancer
GSCALite	Gene Set Cancer Analysis Lite
GSVA	Gene Set Variation Analysis
HDACs	Histone deacetylases
HPA	Human protein atlas
IC50	Half-maximal inhibitory concentration
IFN	Interferon
KIRC	Kidney renal clear cell carcinoma
LAML	Acute myeloid leukemia
LGG	Brain lower-grade glioma
LRRC41	Leucine-rich repeat containing 41
MEM	Minimum essential medium
NAE	NEDD8 activating enzyme
NEDD8	Neuronal precursor cell-expressed developmentally down-regulated protein 8
NEDP1	NEDD8-specific protease 1
PBS	Phosphate-buffered saline
PCPG	Pheochromocytoma and paraganglioma
PSMA	Proteasome 20S subunit alpha
PSMB	Proteasome 20S subunit beta
RBX1/2	RING-box protein 1/2
RCC	Renal cell carcinoma
SARC	Sarcoma
SIRT	Sirtuin
SNV	Single nucleotide variation
SPSB1	SplA/Ryanodine receptor domain and SOCS box containing 1
TCGA	The Cancer Genome Atlas
THCA	Thyroid carcinoma
TME	Tumor microenvironment
UBA3	Ubiquitin-like modifier-activating enzyme 3
UBD	Ubiquitin D
UBE2F/M	Ubiquitin-conjugating enzyme E2 F/M
UCHL3	Ubiquitin C-terminal hydrolase L3
WSB1	WD repeat and SOCS box containing 1
