# Supplementary material for: Evaluating the Role of Neddylation Modifications in Kidney Renal Clear Cell Carcinoma: An Integrated Approach Using Bioinformatics, MLN4924 Dosing Experiments, and RNA Sequencing
Source: Pharmaceuticals (Basel). 2024 May 15;17(5):635. doi: 10.3390/ph17050635 (PMC11125012; doi:10.3390/ph17050635)
Supplement: Supplementary file 1 [file pharmaceuticals-17-00635-s001.zip › Supplement FigureS1-S7.pdf]

Supplementary Material

Figure captions

Figure S1-Widespread genetic mutations in neddylation-related genes. (A) Color bars depicting mRNA expression of neddylation-related genes utilize blue and red to represent decreased and increased expression, respectively. (B) Influence of neddylation-related genes on diverse cellular pathways. Red and purple color bars respectively signify increased or decreased activity of the corresponding cellular pathway.

Figure S2-Comprehensive cluster analysis conducted predicated on neddylation scores. (A) The heatmap encapsulates the segregation of neddylation-related genes into three distinct groups predicated on varying activity scores. Within the heatmap, upregulated gene expression is denoted by a red color, downregulated expression by blue, and relatively stable expression by gray. (B) The violin plot is employed to exhibit the density distribution and distinct differences between each cluster: middle scores (cluster 1 or C1), high scores (cluster 2 or C2), and low scores (cluster 3 or C3). Levels of statistical significance are denoted as follows: *$p<0.05$, **$p<0.01$, and ***$p<0.001$.

Figure S3-The impact of the neddylation score on immune infiltration. (A-D) The relationship between the neddylation score and the infiltration of Tfh, T cell co-stimulation, inflammation-promoting, and CD8 T cells was analyzed and was represented in the scatter diagrams depicted.

Figure S4-Multi-level screening of neddylation-related genes that have specific effects on the prognosis of KIRC. (A) Kaplan-Meier survival curves for 13 significant genes. The plotted graph displays the survival probability over time for two distinctive groups: the high-expression group is represented by an orange line and the low-expression group by a green line. The horizontal axis corresponds to the time in days from the initial diagnosis, while the vertical axis signifies the survival probability. (B) Expression levels of neddylation-related genes in KIRC. (C) The co-expression relationships between PSMB10 and other neddylation-related genes are illustrated using a scatter plot.

Figure S5-Comprehensive investigation into the effects of MLN4924-induced inhibition of neddylation modification on the KIRC phenotype. (A) NEDD8 and PSMB10 Protein Expression Analysis Post-MLN4924 Treatment: Comparative immunofluorescence studies in ACHN and 786-O cell lines illustrate changes in NEDD8 and PSMB10 protein levels following MLN4924 treatment (2 μM; 24h) versus the negative control (NC) group. (B-E) Semi-Quantitative Protein Expression Analysis: Demonstrates downregulation of NEDD8 and PSMB10 expression in both ACHN and 786-O cell lines post-MLN4924 treatment, underscoring the drug's impact on protein regulation. (F) Transwell Migration and Invasion Assay Results Post-MLN4924 Treatment: Comparative analysis of cell migration and invasion abilities in ACHN and 786-O cell lines following MLN4924 (2 μM; 24h) treatment compared to NC groups, indicating diminished capabilities. (G) Statistical Analysis of Transwell Migration Experiments: Aggregated data from three independent experiments showcasing significantly reduced cell migration ability in MLN4924 (2 μM; 24h) treated groups versus NC. (H) Comprehensive Invasion Analysis from Transwell Experiments: Consolidated results from three independent invasion assays reveal a significant reduction in the invasion ability of cells treated with MLN4924 (2 μM; 24h) compared to the NC group. (I) Scratch Assay for Cell Migration Post-MLN4924 Treatment: Evaluates the migration of ACHN and 786-O cell lines at 0, 12, and 24 hours after MLN4924 treatment (2 μM; 24h) relative to the NC group, showing reduced migration speed. (J-K) Quantitative Analysis of Scratch Assays: Statistical analysis from three independent experiments confirms the reduced migration ability of MLN4924 (2 μM; 24h) treated groups versus NC, based on the measured wound closure rate. Statistical significance is

denoted by *p < 0.05, **p < 0.01, ***p < 0.001, ****p < 0.0001.

Figure S6-Comparative RNA sequencing analysis between MLN4924-treated and control groups. (A) Agarose gel electrophoresis of total RNA: This panel displays the integrity assessment of extracted total RNA via agarose gel electrophoresis, providing a visual representation of RNA quality prior to sequencing. (B) Sequencing Data Filtering Process. (C) Differential gene expression volcano plot. Genes with increased expression are shown on the right, while those with decreased expression are on the left. (D) Hierarchical clustering was conducted on all gene expression intensities, and the results were visualized using a heatmap.

Figure S7-Comprehensive analysis of PSMB10 gene expression and functional impact on KIRC cell lines. (A) qRT-PCR analysis comparing PSMB10 expression in ACHN and 786-O cell lines with the normal kidney HK-2 cell line. (B) Evaluation of siRNA-mediated knockdown efficiency in the 786-O cell line using qRT-PCR. (C) Assessment of siRNA (homo-908) knockdown efficiency in the ACHN cell line using qRT-PCR. (D-E) Fluorescence microscopy imaging of ACHN and 786-O Cells transfected with FAM-labeled negative control siRNA. The presence of green fluorescence in the cytoplasm indicates successful transfection. (F) PSMB10 protein expression post-knockdown in ACHN cell line: comparative immunofluorescence analysis between siRNA (homo-908) treated and NC (negative control) groups. (G) Semi-quantitative analysis: PSMB10 immunofluorescence expression differences before and after knockdown in ACHN cell lines. (H) PSMB10 protein expression post-knockdown in 786-O cell line: immunofluorescence comparison between siRNA (homo-908) treated and NC groups. (I) Semi-quantitative analysis: PSMB10 immunofluorescence expression differences before and after knockdown in 786-O cell lines. (J) Comparison of scratch assay results at 0h, 12h, and 24h for ACHN and 786-O cell lines in Si-PSMB10 and NC groups. (K-L) Quantitative analysis derived from three independent replicates of the scratch assay, providing statistical evidence of the migration capacity in Si-PSMB10 versus NC groups. (M) Comparative results from transwell migration and invasion assays for Si-PSMB10 and NC groups in ACHN and 786-O cell lines. (N) Statistical aggregation of data from three independent transwell migration experiments, highlighting differences in migratory behavior between Si-PSMB10 and NC groups. (O) Consolidated data analysis from three separate transwell invasion experiments. *p < 0.05, **p < 0.01, ***p < 0.001, ****p < 0.0001.

Figure S1

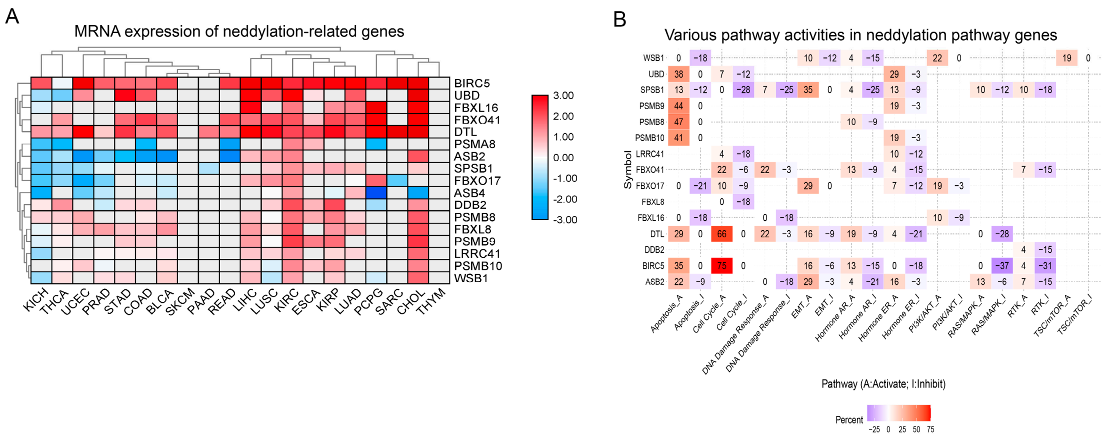

Figure S2

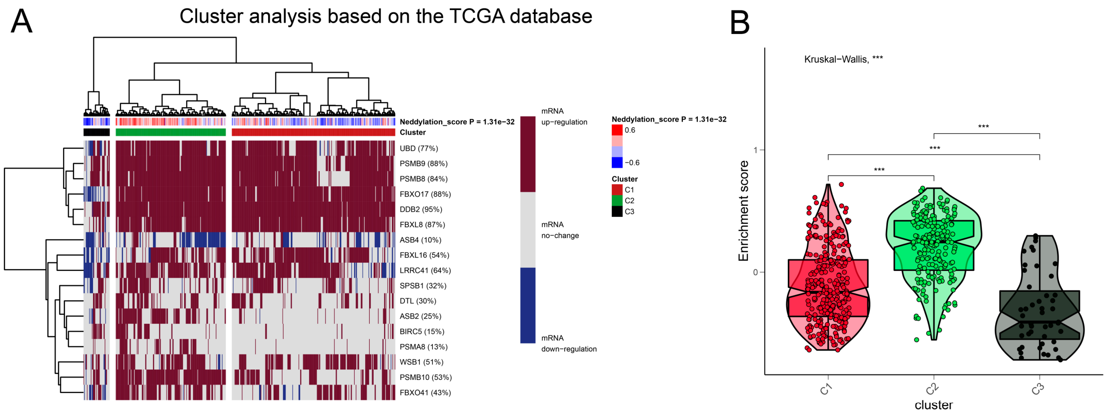

Figure S3

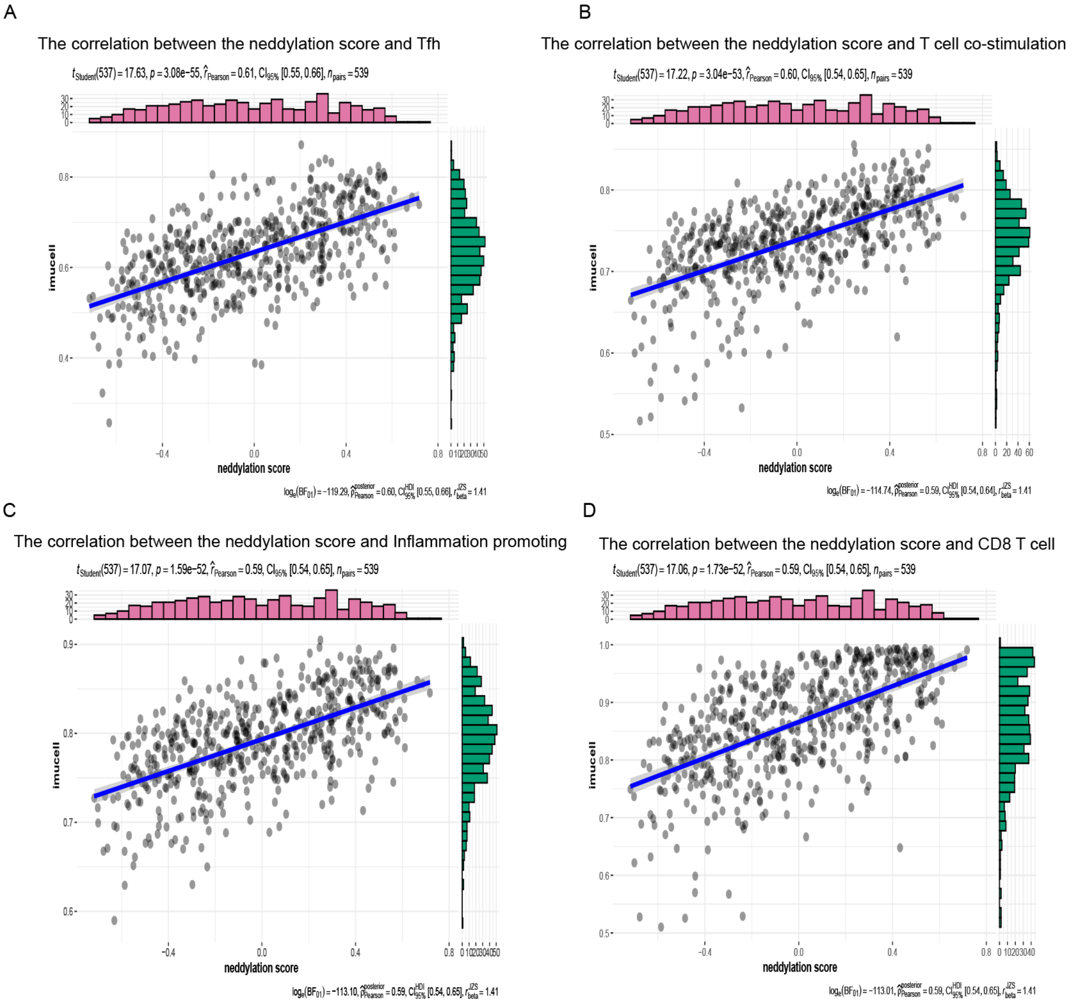

Figure S4

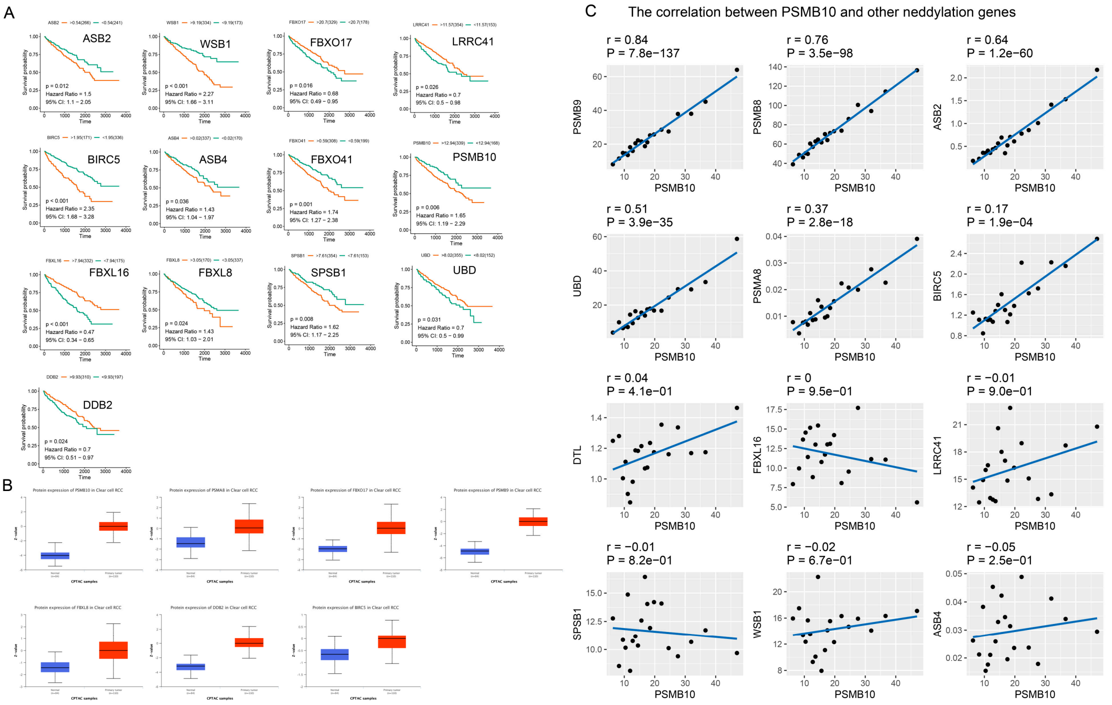

Figure S5

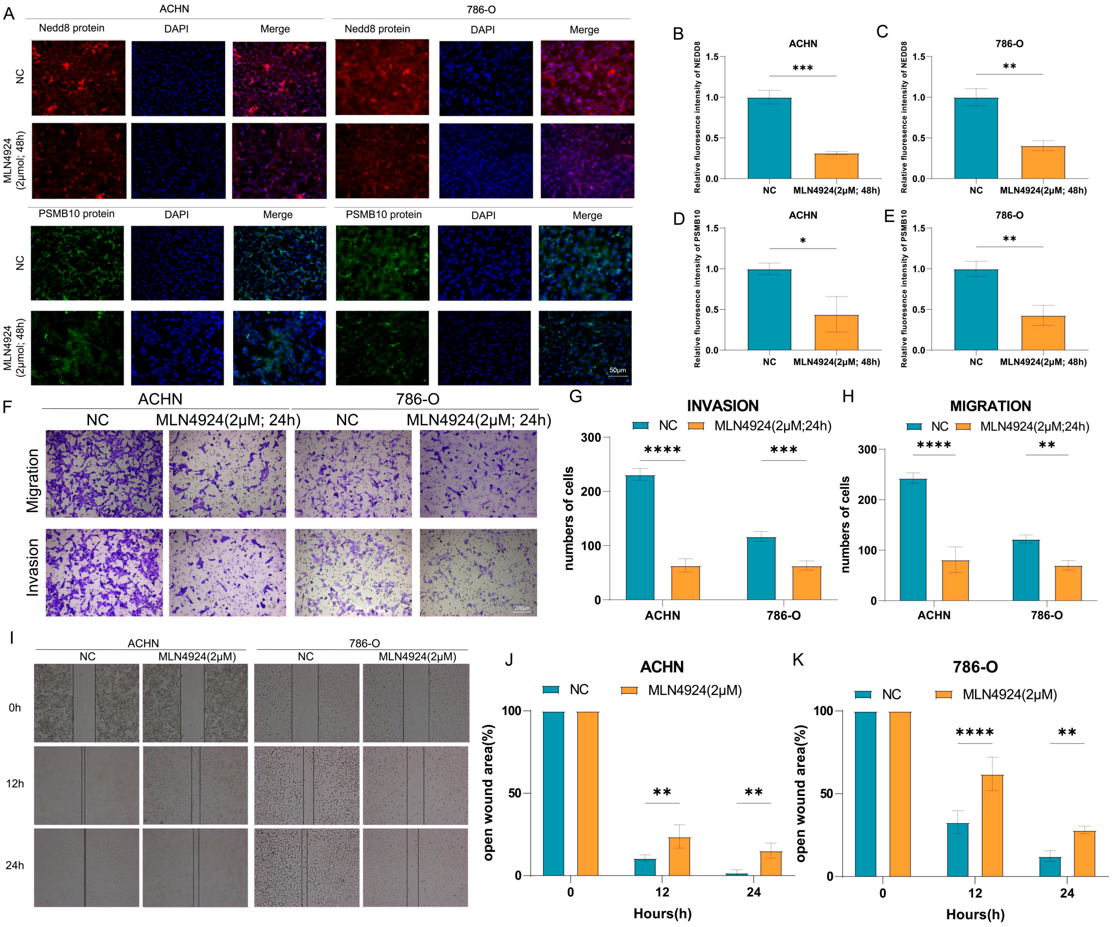

Figure S6

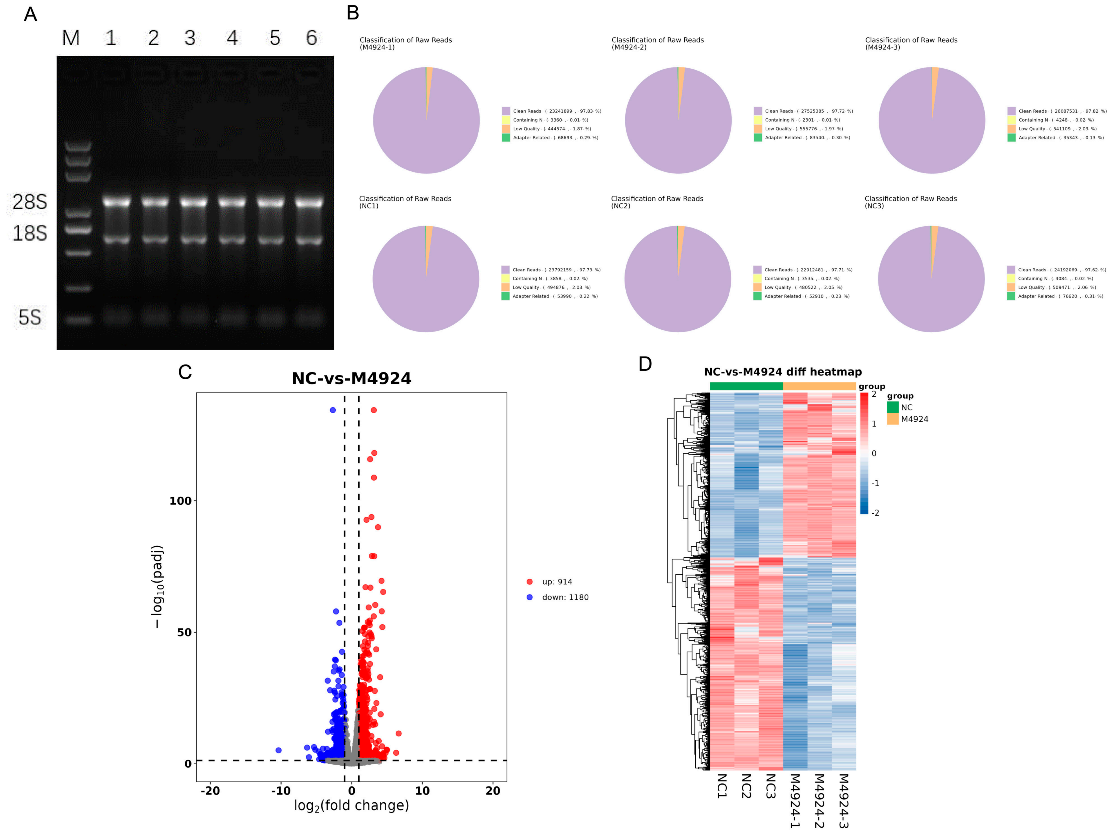
A
M 1 2 3 4 5 6
28S
18S
5S
B
Classification of Raw Reads (M4924-1)
Clean Reads (23241899 , 97.83 %)
Containing N (3360 , 0.01 %)
Low Quality (444574 , 1.87 %)
Adapter Related (68693 , 0.29 %)
Classification of Raw Reads (M4924-2)
Clean Reads (27525385 , 97.72 %)
Containing N (2301 , 0.01 %)
Low Quality (555776 , 1.97 %)
Adapter Related (83540 , 0.30 %)
Classification of Raw Reads (M4924-3)
Clean Reads (26087531 , 97.82 %)
Containing N (4248 , 0.02 %)
Low Quality (541109 , 2.03 %)
Adapter Related (35343 , 0.13 %)
Classification of Raw Reads (NC1)
Clean Reads (23792159 , 97.73 %)
Containing N (3858 , 0.02 %)
Low Quality (494876 , 2.03 %)
Adapter Related (53990 , 0.22 %)
Classification of Raw Reads (NC2)
Clean Reads (22912481 , 97.71 %)
Containing N (3535 , 0.02 %)
Low Quality (480522 , 2.05 %)
Adapter Related (52910 , 0.23 %)
Classification of Raw Reads (NC3)
Clean Reads (24192069 , 97.62 %)
Containing N (4084 , 0.02 %)
Low Quality (509471 , 2.06 %)
Adapter Related (76620 , 0.31 %)
C
NC-vs-M4924
$-\log_{10}(padj)$
100
50
0
-20 -10 0 10 20
$\log_2$(fold change)
up: 914
down: 1180
D
NC-vs-M4924 diff heatmap
group
group
NC
M4924
2
1
0
-1
-2
NC1
NC2
NC3
M4924-1
M4924-2
M4924-3

Figure S7

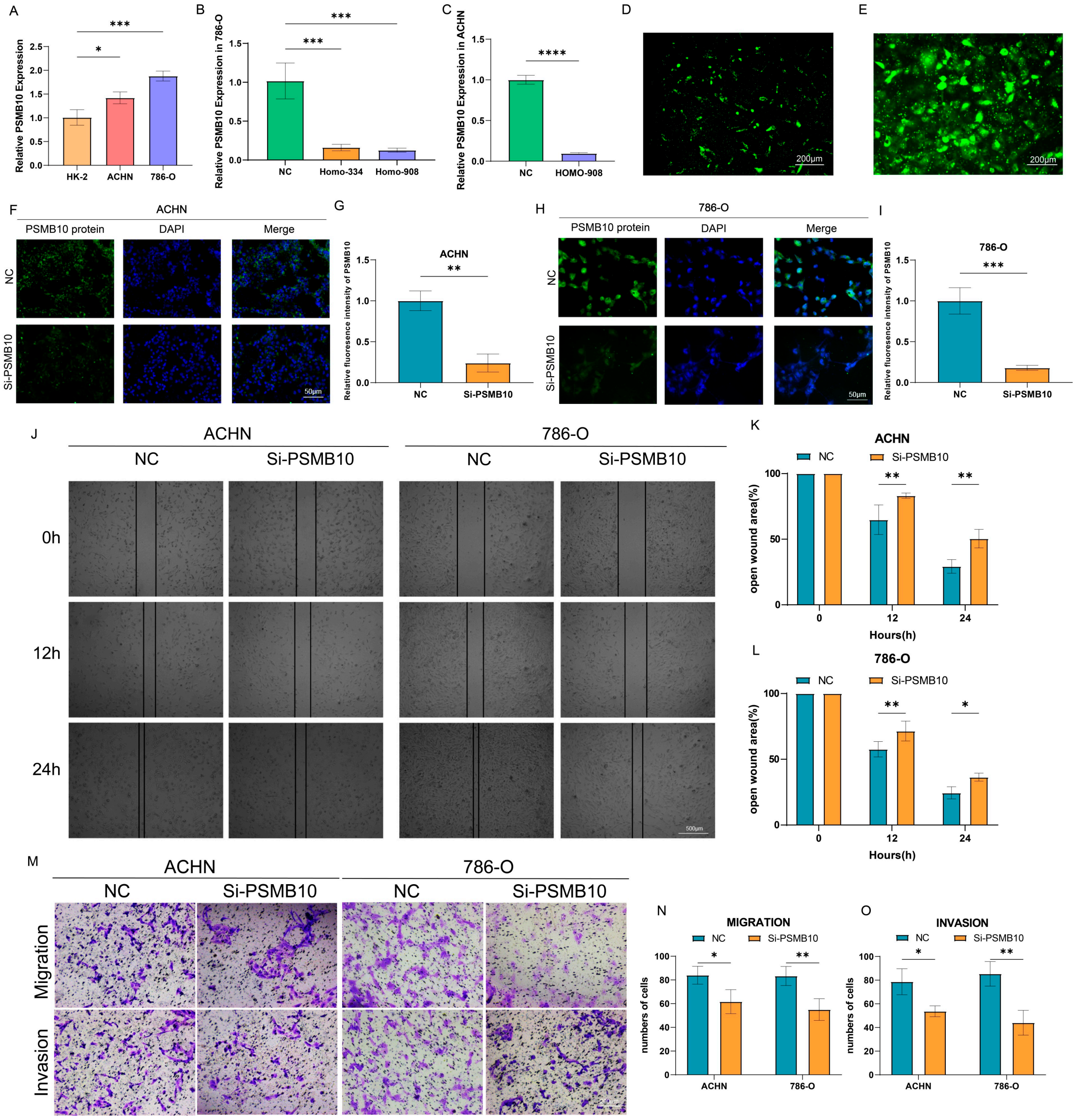
A
Relative PSMB10 Expression
HK-2
ACHN
786-O
B
Relative PSMB10 Expression in 786-O
NC
Homo-334
Homo-908
C
Relative PSMB10 Expression in ACHN
NC
HOMO-908
D
E
200μm
F
ACHN
PSMB10 protein
DAPI
Merge
NC
Si-PSMB10
50μm
G
ACHN
Relative fluoresence intensity of PSMB10
Si-PSMB10
H
786-O
I
J
0h
12h
24h
500μm
K
open wound area(%)
Hours(h)
L
M
Migration
Invasion
N
MIGRATION
numbers of cells
O
INVASION
